# Multi-Domain Feature Fusion and Channel Attention in an Inception-Based Architecture for Motor Imagery EEG Decoding

**DOI:** 10.3390/brainsci16090910

**Published:** 2026-08-26

**Authors:** Sitong Liu, Guangyu Zhang, Cunwei Wu, Wenjia Li, Yanan Zhang, Boao Wei, Yunfei Ma, Hongkai Wang, Xiaohong Huang

**Affiliations:** 1 School of Artificial Intelligence, North China University of Science and Technology, Tangshan 063210, China; 2 Department of Mechanical Engineering, North China University of Science and Technology, Tangshan 063210, China; 3 Hebei Key Laboratory of Industrial Intelligent Perception, Tangshan 063210, China

**Keywords:** electroencephalography (EEG), motor imagery, SE-EEG-inception, multi-domain feature extraction

## Abstract

**Highlights:**

**What are the main findings?**

- Evaluated under a rigorous intra-subject (subject-dependent) cross-validation protocol across 292 individuals, the SE-EEG-Inception model achieved a mean 4-class accuracy of 89.4%. Binary classification accuracies reached 88.3% for left vs. right arm and 90.0% for left vs. right leg.
- These results reveal that the proposed framework can distinguish motor imagery patterns across different limb regions and effectively classify predictive EEG features between symmetric limbs performing identical imagined movements.

**What are the implications of the main findings?**

- Non-invasive EEG can be used to identify distinct motor imagery patterns and classify predictive signals between symmetric limbs (e.g., left vs. right arm, left vs. right leg), laying the foundation for developing multi-limb motor imagery brain–computer interface systems.
- These findings may support future applications in intelligent prosthetic control and neurorehabilitation.

**Abstract:**

**Background/Objectives**: Existing motor imagery (MI) EEG decoding is often limited by small datasets, affecting generalization reliability. This study aims to robustly decode multi-limb MI intentions. **Methods**: We collected an MI-EEG dataset from 292 participants (242 young adults, 50 older adults) performing left/right-arm and left/right-leg imagery. After extracting time-, frequency-, and channel correlation features, we proposed an SE-EEG-Inception model for classification. **Results**: Evaluated under a strict intra-subject cross-validation protocol, the model achieved a mean 4-class accuracy of 89.4%. For binary tasks, accuracies reached 88.3% (left vs. right arm) and 90.0% (left vs. right leg). **Conclusions**: The model successfully distinguishes predictive EEG features across different and symmetric limbs. Crucially, this high classification performance demonstrates data-driven predictive utility rather than mechanistic proof of neural differences, providing an offline proof-of-concept for multi-limb BCI control.

## 1. Introduction

Brain–computer interface technology establishes a communication pathway between the brain and external devices, showing great potential in rehabilitation medicine, assistive control, neurorehabilitation, and intelligent human–computer interaction. Motor imagery (MI) is one of the core paradigms in BCI research. During MI tasks, users imagine specific movements of certain body parts, such as the left arm, right arm, left leg, or right leg, and the corresponding neural activities are reflected in sensorimotor EEG rhythms. Compared with BCI paradigms that rely on external stimulation or actual movement execution, MI-based BCI is more closely associated with instructed motor imagery and active neural regulation, making it valuable for neurorehabilitation training, assistive interaction, and motor function recovery [1,2].

However, high-quality MI-EEG datasets remain limited. Many existing studies are based on relatively small datasets or single-population settings, which may limit model generalizability, especially in multi-class MI classification [3,4]. In addition, conventional MI-EEG decoding methods commonly rely on temporal complexity and spectral rhythm representations, but they may not fully characterize the nonlinear spatiotemporal dependencies embedded in EEG signals. Therefore, an effective MI-EEG decoding model should not only extract discriminative multi-scale representations but also dynamically recalibrating channel-wise feature responses.

Recent MI-EEG decoding studies have primarily advanced along three distinct technical routes closely related to our work: multi-branch convolutional architectures [5,6], multi-domain feature fusion [7,8] and channel attention enhancement [9,10]. To illustrate these advancements and their current limitations, several recent studies serve as representative examples within these categories.

Representing the multi-branch route, Qin et al. [11] proposed models such as ETCNet, and Lou et al. [12] developed EEG-DBNet. These models learn representations end-to-end from raw signals via parallel convolutional branches, which enable multi-scale signal decomposition without manual feature engineering. Nevertheless, they seldom incorporate explicit neurophysiological priors, leading to limited feature interpretability. Regarding feature fusion methods [7,8], most simply concatenate time, frequency, or spatial-domain features, lacking adaptive weighting for heterogeneous feature sources. For instance, Chen et al. [13] proposed graph-based spatial fusion approaches such as MST-DGCN, which focus on inter-channel topological dependencies; yet, most graph-based MI-EEG models are validated on small homogeneous datasets, with limited verification on large-scale age-diverse cohorts.

Therefore, this study constructs a large-scale private four-class MI-EEG dataset comprising 292 participants and proposes the use of the SE-EEG-Inception model for robust multi-class MI-EEG decoding. Based on the extracted multi-domain EEG features, the model encodes multi-domain representations through multi-scale Inception branches and adaptively recalibrates the importance of different channel features using the SE attention mechanism. This design enhances motor intention-related information, thereby improving the classification performance of multi-class motor imagery.

The core objective of this study is to improve the discriminability of MI-related EEG patterns by integrating multi-scale convolutional feature extraction, channel-wise attention, and multi-dimensional EEG feature representation, while maintaining a compact and generalizable model structure. Under an intra-subject (subject-dependent) evaluation protocol, the proposed SE-EEG-Inception architecture demonstrates highly competitive and personalized classification performance. The model effectively adapts to individual EEG characteristics, providing robust decoding capabilities tailored to each user.

The main contributions of this work are summarized as follows:

(1) An SE-EEG-Inception model is proposed by combining multi-scale convolutional feature extraction with channel-wise attention. This coordinated encoding mechanism enables the model to capture complementary EEG representations at different scales while adaptively emphasizing task-relevant neural information.

(2) Time-domain, frequency-domain, and spatial-domain EEG features are extracted and analyzed in relation to physiological characteristics. Redundant feature components are removed to enhance the model’s ability to distinguish ambiguous motor imagery categories.

(3) A rigorous intra-subject evaluation pipeline is established on a large-scale, highly heterogeneous cohort (*n* = 292). By achieving stable personalized classification across distinct demographic groups, this study provides a robust methodological baseline and validates the feasibility of decoding multi-limb motor intentions (including symmetric limbs).

## 2. Materials and Methods

**Figure 1:**
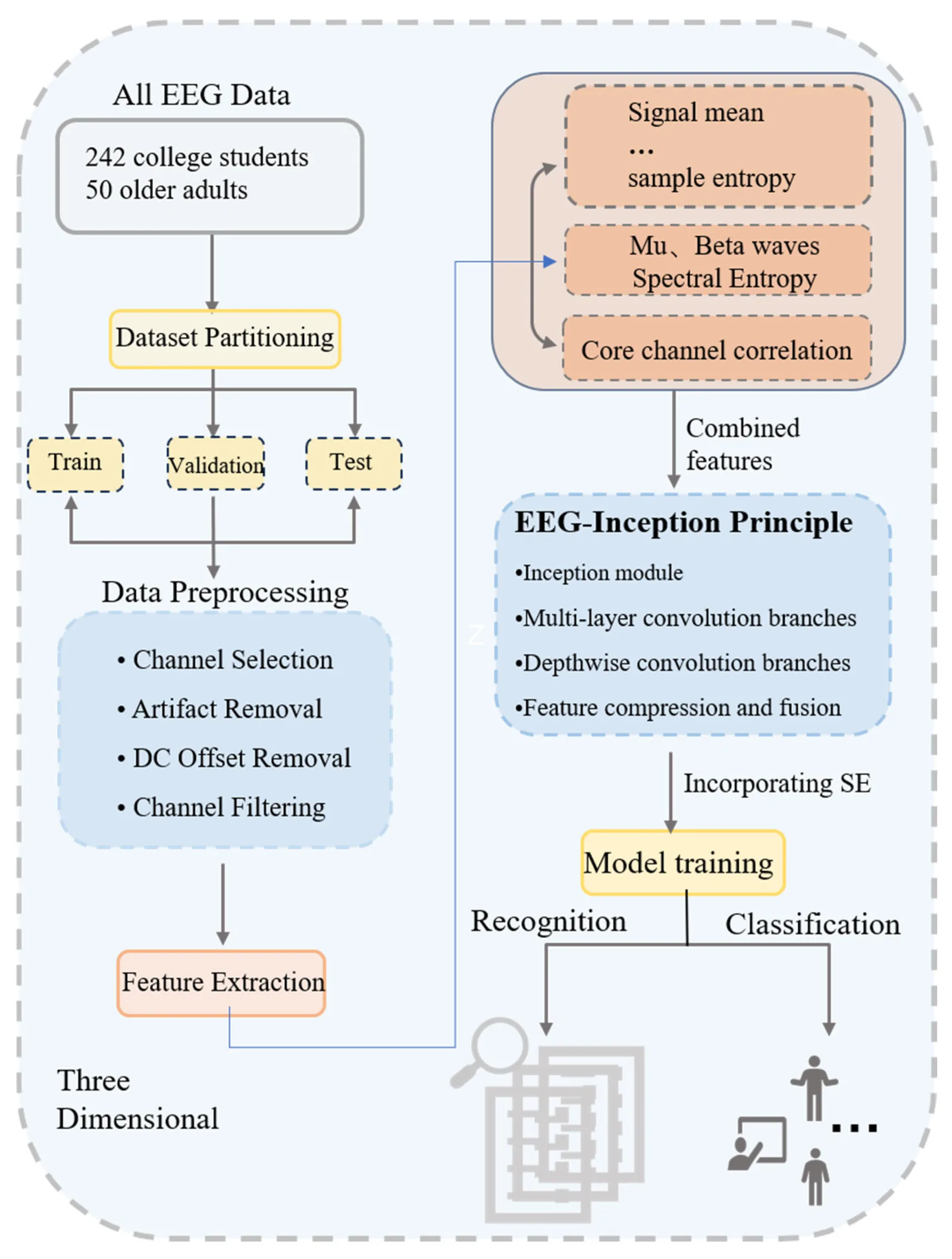
Overall technical framework of the proposed SE-EEG-Inception method.

The complete technical framework of this study is illustrated in Figure 1. The overall workflow begins with the collection of age-stratified MI-EEG data, followed by dataset partitioning, data preprocessing, multi-domain feature extraction, and model training. During preprocessing, motor-cortex-related EEG channels are selected, and artifact removal, DC offset correction, and bandpass filtering are performed to improve signal quality. Subsequently, time-domain, frequency-domain, and spatial-domain features are extracted and integrated to construct the input representation for the proposed model.

### 2.1. EEG Data Collection

We adopt the Classification Easy-Difficult Scale (CE-DS) paradigm first proposed in this study to grade the difficulty of four categories of motor imagery tasks. This age-stratified CE-DS paradigm covers four basic motor imagery (MI) tasks of large-limb imagination, including left-arm, right-arm, left-leg and right-leg imagery. The paradigm is designed to establish a standardized experimental setup adaptive to characteristics of different age groups, so as to evaluate the robustness of MI-EEG decoding algorithms across diverse subject cohorts.

To explicitly introduce real-world data heterogeneity (e.g., natural variations in environment, baseline impedance, and differing trial counts) rather than conducting a controlled cohort comparison, this study integrated two independent MI-EEG datasets collected from different sites.

This study integrated two MI-EEG datasets collected from young adults and older adults under comparable task settings (see Table 1). The young-adult dataset was collected at Inner Mongolia University and included 242 young-adult participants. Four MI tasks were designed, including left-arm, right-arm, left-leg, and right-leg motor imagery. Each participant completed 58 trials for each task, resulting in 232 trials per participant and 56,144 trials in total. EEG signals were recorded using a 64-channel EEG acquisition system. Each trial lasted 5 s, including a 4 s motor imagery period, and the sampling rate was 1000 Hz. The older-adult cohort data was collected from Xinglong New Village in Tongliao City and included 50 older-adult participants. The same four MI tasks were adopted. Each participant completed 82 trials for each task, resulting in 328 trials per participant and 16,400 trials in total. EEG signals were also recorded using a 64-channel EEG acquisition system. Each trial lasted 5 s, including a 4 s motor imagery period, and the sampling rate was 1000 Hz. Overall, the combined MI-EEG dataset initially comprised 292 participants and 72,544 nominal trials. To ensure the validity of the recorded EEG signals, all recruited participants were self-reported healthy individuals who were naive to BCI systems, possessed normal or corrected-to-normal vision, and were capable of understanding and executing the motor imagery tasks. Because the primary objective of this study was generalized algorithmic development rather than clinical profiling, exhaustive clinical metadata (such as formalized cognitive screening, handedness, or medication logs) were not systematically recorded.

Furthermore, Figure S1 illustrates the detailed recruitment, quality control, and subsequent data partitioning process. Rigorous quality control was enforced, wherein trials exhibiting a mean peak-to-peak amplitude > 50 µV across channels were entirely discarded. Consequently, 0 participants were entirely excluded due to excessive recording noise or poor task compliance. The final effective sample size entered into all subsequent analyses comprised 292 participants, yielding a total of 72,278 highly reliable, usable trials, which were then divided into an independent test set (19.3%) and a development set for 5-fold cross-validation (80.7%).

The experimental configuration followed the preprocessing and feature extraction pipeline described in Section 2.3 and Section 2.4, including channel selection, ICA-based artifact removal, DC offset removal, bandpass filtering at 8–30 Hz, and multi-domain feature extraction. Classification accuracy and macro-averaged F1-score were used as the main evaluation metrics. All data augmentation operations were strictly applied only to the training set after data partitioning. No augmented samples derived from validation or test trials were used during training, thereby preventing any information leakage. The detailed dataset partitioning algorithm and cross-validation strategy are explicitly described in Section 2.6.

### 2.2. Experimental Paradigm

To ensure task compliance and reduce intra-subject variability, the experimental procedure consisted of a pre-task familiarization session and a formal recording session, both conducted under the recording configurations described in Section 2.1. To ensure high-quality data acquisition across both sites, strict and standardized procedural protocols were maintained. EEG signals were recorded using a Compumedics Neuroscan SynAmps RT amplifier with a 64-channel Quik-Cap (Ag/AgCl electrodes). The 64-channel EEG acquisition device was manufactured by Brain Products GmbH, Gilching, Germany. The linked mastoids (M1/M2) served as the reference, and AFz served as the ground electrode. Electrode impedances were strictly maintained below 5 kΩ throughout the recording. Data acquisition was performed using CURRY 8 software in an electromagnetically shielded and sound-attenuated room. Visual cues were delivered via a 24-inch LCD monitor running E-Prime 3.0. Furthermore, operators at both sites underwent a standardized training program focused on impedance minimization and consistent participant instruction.

**Figure 2:**
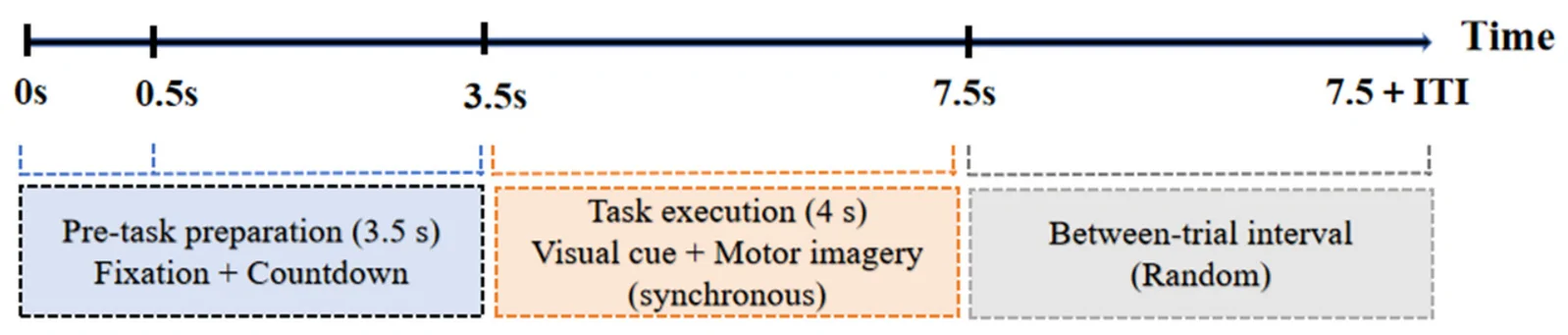
Schematic illustration of the experimental trial timeline.

The formal recording session adopted a visually cued MI paradigm. To strictly prevent the classification model from exploiting visually evoked potentials (VEPs) or differential eye movements, all task-specific visual cues presented during the 4 s imagery period were rigorously matched in terms of luminance, contrast, spatial frequency, and physical dimensions. Furthermore, the presentation order of the four MI classes was fully randomized across trials to prevent sequence-specific cognitive confounds. To eliminate any descriptive ambiguity regarding the temporal sequence, the exact duration, structural purpose, and chronological order of each phase within a single trial are systematically illustrated in a dedicated timeline schematic (Figure 2). Upon cue presentation, participants were instructed to mentally simulate the indicated movement without any overt physical execution. Before cue onset, a “3-2-1” countdown was presented to guide preparation and attention focusing. During the experiment, participants were required to remain physically still and avoid unnecessary body or limb movement.

**Figure 3:**
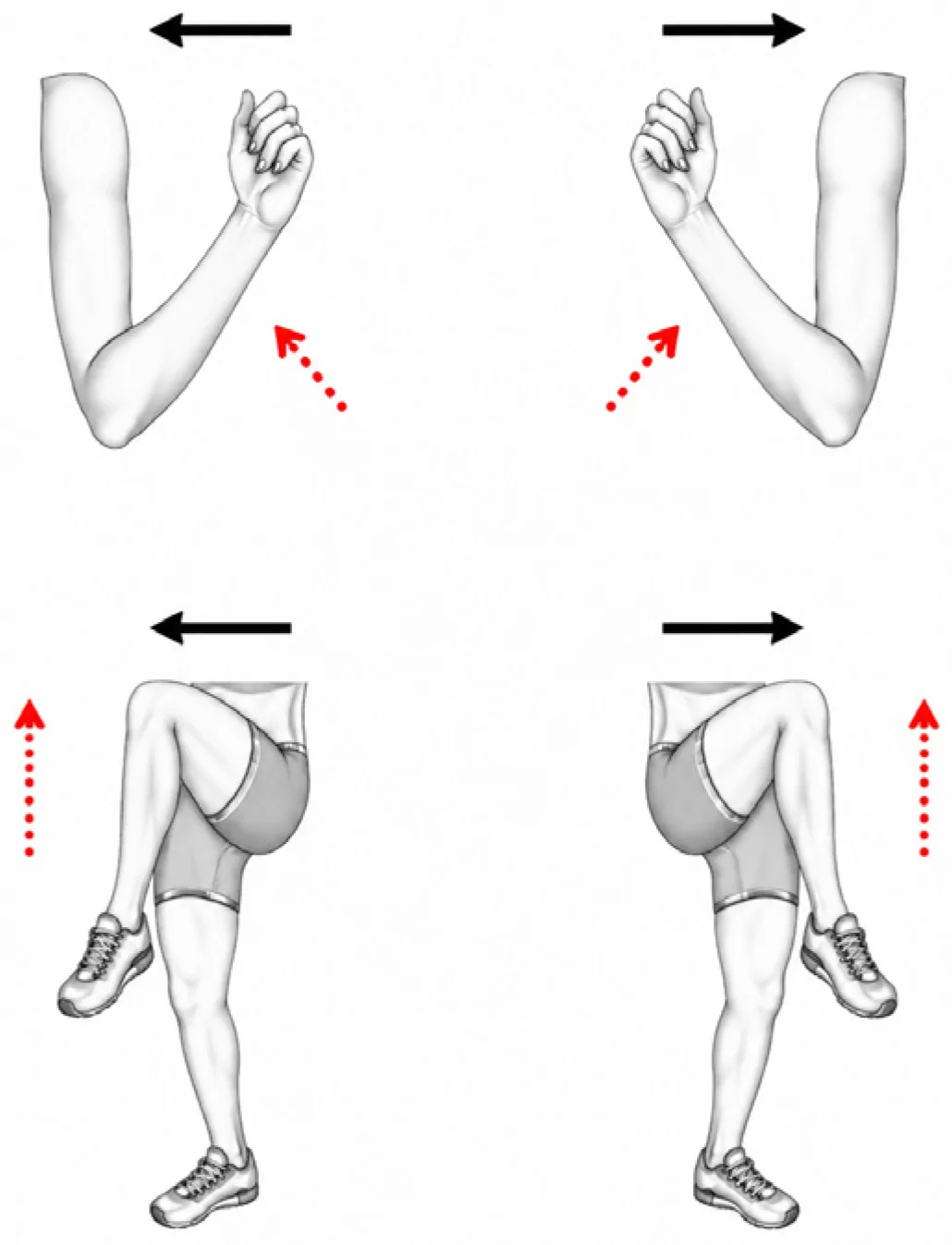
Illustration of the motor imagery task cues.

Before EEG recording, all participants underwent a standardized familiarization procedure. The experimenter explained the concept of motor imagery (MI), introduced the four MI task categories, and demonstrated the corresponding task requirements. Participants then practiced the tasks without overt physical execution until they fully understood the instructions and could perform the imagery procedure consistently. This step was intended to improve task compliance and reduce variability during the formal recording session. The visual cues used in the experiment are illustrated in Figure 3.

The complete trial timeline comprised five distinct chronological phases: a baseline fixation period, a 3 s pre-cue countdown (“3-2-1”), a 4 s motor imagery period executed synchronously with the visual cue presentation, and a randomized inter-trial interval. Randomized inter-trial intervals were used to reduce anticipatory effects. Specific task allocations and trial counts for the two age groups are provided in Section 2.1. All subsequent data processing followed the standardized pipeline described in Section 2.3 and Section 2.4.

### 2.3. Data Preprocessing

For EEG decoding models trained on low signal-to-noise ratio (SNR) data, improving signal quality and reducing artifact contamination are essential preprocessing steps. To ensure computational reproducibility and strictly prevent cross-partition data leakage, the preprocessing pipeline was executed independently at the single-trial level within segregated data splits. Initially, an automated amplitude threshold was applied to reject heavily contaminated trials; specifically, any trial exhibiting a mean peak-to-peak amplitude > 50 µV across channels was entirely discarded. For retained trials, Independent Component Analysis (FastICA algorithm) was fitted separately to each 5 s epoch. To ensure adequate matrix conditioning given the short epoch duration, dimensionality was reduced to 80% of the available channels via Principal Component Analysis (PCA) prior to ICA. An automated component selection criterion identified and excluded a maximum of two components exhibiting high correlation with frontal EOG-proxy channels. Following artifact removal, the DC offset of each trial was eliminated by subtracting its temporal mean. A 4th-order Butterworth bandpass filter (8–30 Hz) was then applied. Zero-phase distortion was maintained using forward-backward filtering (SciPy filtfilt with default odd-extrapolation edge padding). The original sampling rate of 1000 Hz was retained without downsampling. By mathematically confining all data-dependent operations to individual trials, this pipeline strictly guarantees the absence of information leakage between training, validation, and testing partitions.

Before preprocessing, each EEG trial was represented as follows:

(1) $$X_{i}\in R^{C\times T},\;y_{i}\in \{1,2,\ldots ,K\}$$

where $X_{i}$ denotes the i-th EEG trial, C is the number of EEG channels, T is the number of time samples, $y_{i}$ is the corresponding class label, and K is the number of motor imagery classes.

**Figure 4:**
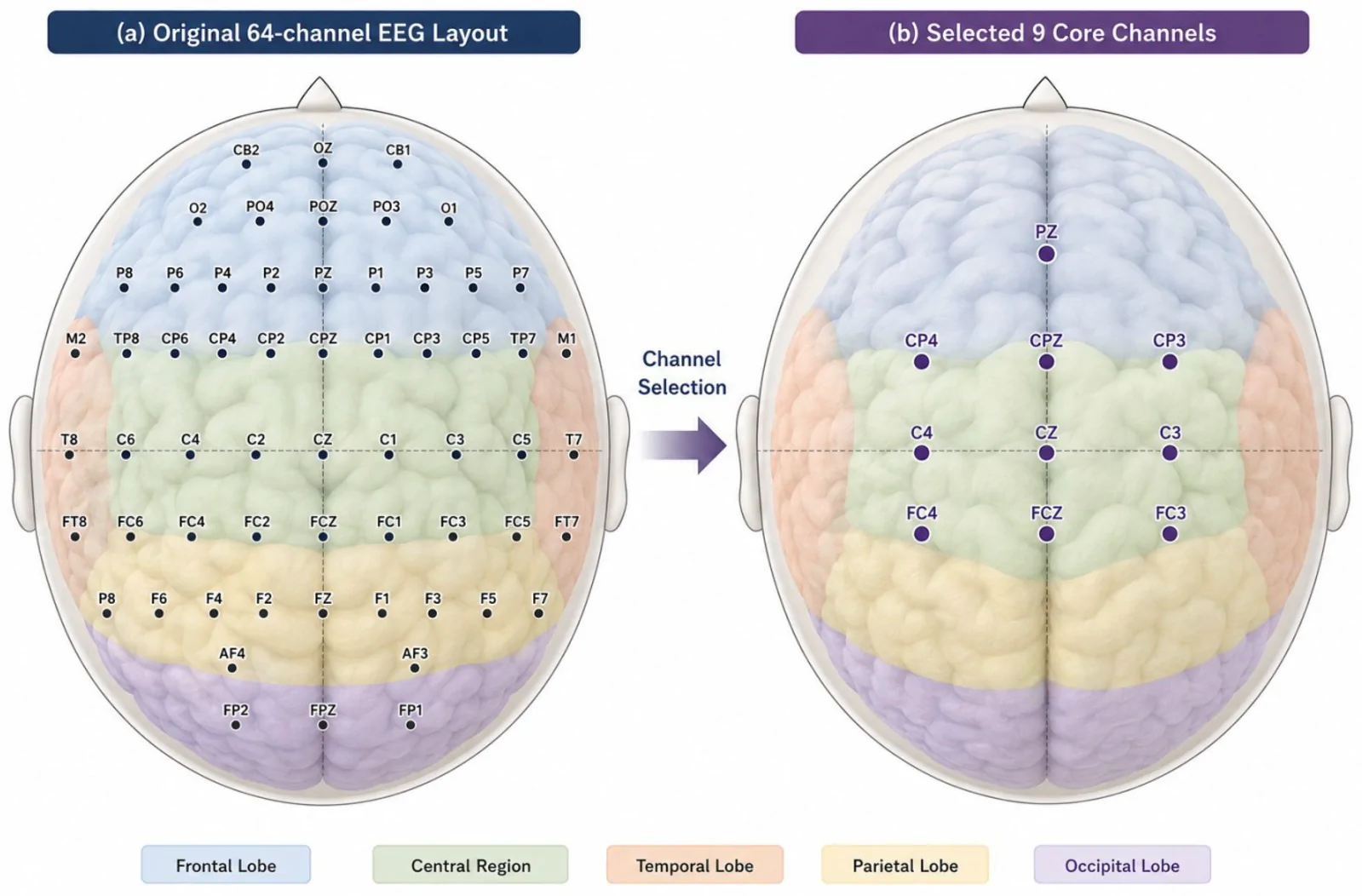
Spatial distribution of the selected EEG channels.

Based on the neural mechanisms of motor imagery, 10 motor-cortex-related EEG channels were selected from the original 64-channel montage, including C3, C4, CP3, CP4, Cz, FC3, FC4, FCz, CPz, and Pz. Among them, C3, C4, CP3, CP4, and Cz were selected as primary motor-related channels, while FC3, FC4, FCz, CPz, and Pz were included to supplement information from adjacent frontal-central, central-parietal, and parietal regions [14]. Although posterior channels (e.g., Pz, CPz) were included to capture broader spatial contexts, the subsequent 8–30 Hz bandpass filtering specifically isolates rolandic sensorimotor rhythms (Mu and Beta dynamics), effectively minimizing the influence of low-frequency visually evoked transients associated with the continuous cue presentation. The spatial distribution of the selected channels is illustrated in Figure 4.

The channel selection process can be formulated as follows:

(2) $$X_{i}^{s}=SX_{i}$$

where $S\in R^{C_{s}\times C}$ denotes the channel selection matrix, $C_{s}=10$ is the number of selected core channels, and $X_{i}^{s}$ is the EEG signal after channel selection.

For each selected EEG channel within each trial, DC offset removal was performed by subtracting the temporal mean from the original signal:

(3) $${\tilde{x}}_{c}\left(t\right)=x_{c}\left(t\right)-\frac{1}{T}\sum_{t=1}^{T}x_{c}\left(t\right)$$

where $x_{c}\left(t\right)$ denotes the original signal of channel (c) at time t, and ${\tilde{x}}_{c}\left(t\right)$ denotes the signal after DC offset removal.

Subsequently, an 8–30 Hz bandpass filter was applied to retain the Mu and Beta rhythm components associated with motor imagery [15]:

(4) $${\hat{x}}_{c}\left(t\right)=B_{8-30}\left({\tilde{x}}_{c}\left(t\right)\right)$$

where $B_{8-30}\left(\cdot \right)$ denotes the 8–30 Hz bandpass filtering operation, which preserves the Mu and Beta rhythm components associated with motor imagery.

### 2.4. Multi-Domain Feature Extraction

To obtain compact, relevant, and non-redundant EEG representations, complementary features were extracted from three domains: the time domain, frequency domain, and spatial domain. These features were designed to characterize both linear and nonlinear information related to motor imagery (MI). Specifically, time-domain features, including the mean, standard deviation, peak-to-peak amplitude, skewness, and sample entropy, were used to describe signal amplitude variation, statistical distribution, and nonlinear complexity. For nonlinear complexity quantification, sample entropy is adopted rather than approximate entropy or multiscale entropy. Approximate entropy suffers from self-matching bias, while multiscale entropy may yield unstable estimates on short single-trial MI-EEG segments due to its coarse-graining operation. Sample entropy can produce stable and unbiased complexity measurements on finite-length trial-level EEG data, which fits our feature-extraction scenario. Frequency-domain features, including Mu-band power, Beta-band power, and spectral entropy, were used to characterize sensorimotor rhythm activities associated with MI. Spatial-domain features were calculated using Pearson correlation coefficients to describe the functional coupling strength among the selected motor-cortex-related EEG channels [16].

Among the time-domain descriptors, Peak-to-peak amplitude describes the amplitude fluctuation range of EEG signal segments:

(5) $$A_{pp}=max\left(x\right)-min\left(x\right)$$

where x denotes the segment-level EEG signal vector.

Skewness reflects the asymmetry of signal amplitude distribution:

(6) $$S=\frac{n}{\left(n-1\right)\left(n-2\right)}\sum_{i=1}^{n}{\left(\frac{x_{i}-\bar{x}}{\sigma }\right)}^{3}$$

where n is the number of sampling points, $\bar{x}$ is the signal mean, and σ is the signal standard deviation.

Sample entropy was used to quantify the nonlinear complexity of EEG signals [17]:

(7) $$SampEn\left(m,r,N\right)=-ln\frac{A^{m+1}\left(r\right)}{A^{m}\left(r\right)}$$

where m is the embedding dimension (set to 2), r is the similarity tolerance (set to 0.2 multiplied by the standard deviation of the time series), N is the length of the time series (3000 points), and $A^{m}\left(r\right)$ and $A^{m+1}\left(r\right)$ represent the matching probabilities of template vectors with dimensions m and m+1, respectively.

Frequency-domain features were calculated based on the power spectral density (PSD), which was estimated via Welch’s method utilizing a 256-point (0.256 s) Hanning window with a 50% overlap:

(8) $$PSD_{c}\left(f\right)=\frac{1}{T}{\left|\sum_{t=1}^{T}x_{c}\left(t\right)e^{-j2\pi ft/T}\right|}^{2}$$

where $PSD_{c}\left(f\right)$ denotes the estimated power spectral density of channel c at frequency f.

The Mu-band and Beta-band powers of channel (c) were obtained by summing the PSD values within the corresponding frequency ranges:

(9) $$P_{c}^{\mu }=\sum_{f=8}^{12}PSD_{c}\left(f\right)$$

(10) $$P_{c}^{\beta }=\sum_{f=13}^{30}PSD_{c}\left(f\right)$$

where $P_{c}^{\mu }$ and $P_{c}^{\beta }$ denote the Mu-band and Beta-band power of channel c, respectively.

Spectral entropy was further calculated from the normalized power distribution:

(11) $$p_{c}\left(f\right)=\frac{PSD_{c}\left(f\right)}{\sum_{f\in \Omega }PSD_{c}\left(f\right)}$$

(12) $$H_{c}=-\sum_{f\in \Omega }p_{c}\left(f\right)logp_{c}\left(f\right)$$

where Ω denotes the selected frequency range, $p_{c}\left(f\right)$ is the normalized spectral power distribution, and $H_{c}$ represents the spectral entropy of channel c. These metrics reflect stable discriminative spectral characteristics captured by the classifier rather than direct Event-Related Desynchronization (ERD) or Synchronization (ERS).

The general functional coupling between any two channels can be described using the Pearson correlation coefficient:

(13) $$\rho_{c_{1},c_{2}}=\frac{\sum_{t=1}^{T}\left(x_{c_{1}}\left(t\right)-\mu_{c_{1}}\right)\left(x_{c_{2}}\left(t\right)-\mu_{c_{2}}\right)}{\sqrt{\sum_{t=1}^{T}{\left(x_{c_{1}}\left(t\right)-\mu_{c_{1}}\right)}^{2}}\sqrt{\sum_{t=1}^{T}{\left(x_{c_{2}}\left(t\right)-\mu_{c_{2}}\right)}^{2}}}$$

where $\rho_{c_{1},c_{2}}$ denotes the Pearson correlation coefficient between two EEG channels, reflecting the channel-wise signal correlation between different motor-cortex-related regions.

Specifically for spatial-domain feature extraction in this study, we computed the signal correlation between each selected motor channel and the central reference channel (Cz):

(14) $$R_{c}=\rho (x_{c},x_{ref})=\frac{\sum_{t=1}^{T}(x_{c}(t)-\mu_{c})(x_{ref}(t)-\mu_{ref})}{\sqrt{\sum_{t=1}^{T}(x_{c}(t)-\mu_{c}{)}^{2}\sum_{t=1}^{T}(x_{ref}(t)-\mu_{ref}{)}^{2}}}$$

where $R_{c}$ represents the channel-wise signal correlation feature of channel $c\;\mathrm{and}\;\rho_{c}$, Cz denotes the Pearson correlation coefficient between channel c and the reference channel Cz.

Finally, the extracted features were integrated into a fused feature matrix for each sample:

(15) $$F_{i}=\left[F_{i}^{time},F_{i}^{freq},F_{i}^{spatial}\right]\in R^{D\times c_{s},}$$

(16) $$D=D_{time}+D_{freq}+D_{spatial}=5+3+1=9$$

where $F_{i}$ denotes the fused feature matrix of the i-th sample, D is the total number of feature types, and $c_{s}$ is the number of selected EEG channels.

**Figure 5:**
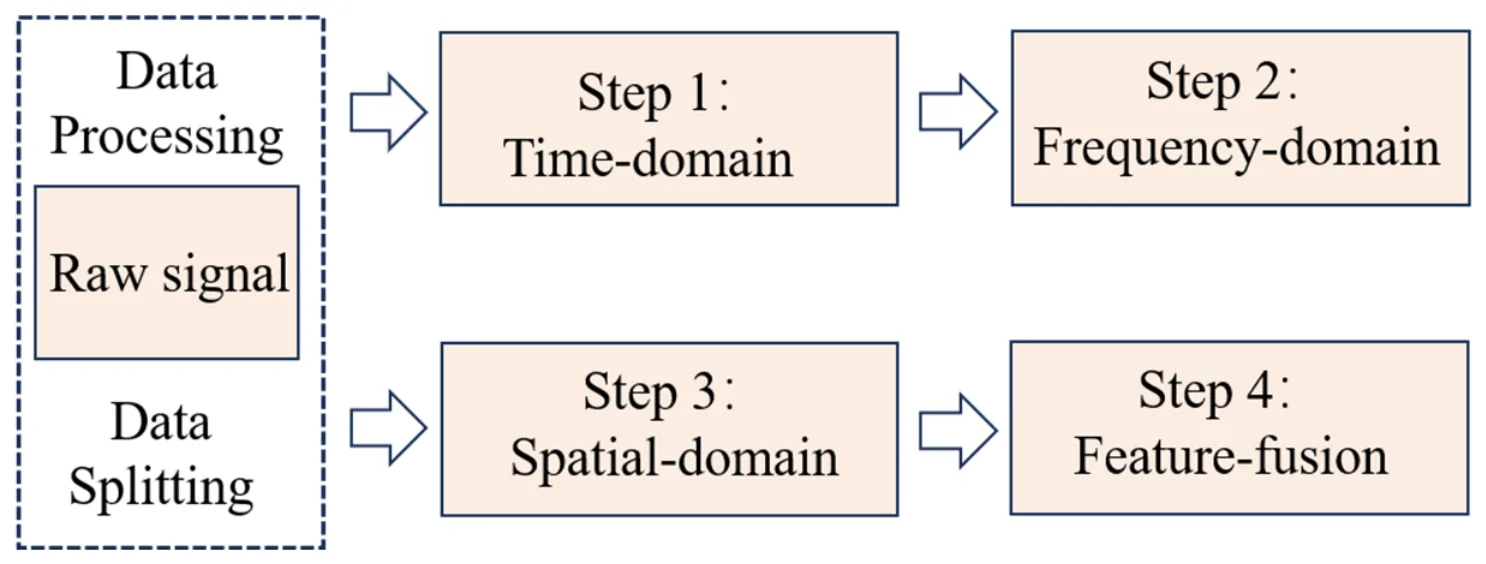
Feature extraction and multi-domain feature fusion pipeline.

Figure 5 illustrates the extraction and fusion process of the multi-domain EEG features. After preprocessing, time-domain features were first extracted to describe signal amplitude variation and nonlinear complexity. Frequency-domain features were then calculated to characterize Mu and Beta rhythm activities related to motor imagery. Spatial-domain features were further obtained by computing inter-channel correlations among the selected sensorimotor-related channels. Finally, the extracted time-domain, frequency-domain, and spatial-domain features were fused into a unified feature representation, forming a three-dimensional feature tensor for subsequent SE-EEG-Inception model training. Prior to model training, the 9 heterogeneous multi-domain features were standardized using Z-score normalization. Consequently, the originally positive absolute band powers were rescaled into centered distributions containing negative values. To strictly prevent data leakage, scaling parameters (mean and standard deviation) were computed exclusively on the training set and subsequently applied to the validation and test sets.

To evaluate the descriptive neurophysiological features and strictly prevent pseudoreplication, statistical inferences were conducted at the participant level. For each participant, trial-level feature values were averaged within each task condition, yielding independent observations (*n* = 292). Data normality was assessed using the Shapiro–Wilk test. Differences across the four motor imagery tasks were evaluated using repeated-measures ANOVA (for normally distributed data) or the Friedman test (for non-normally distributed data). Post hoc pairwise comparisons were performed using paired *t*-tests or Wilcoxon signed-rank tests, respectively. To strictly control the false discovery rate (FDR), all pairwise *p*-values were adjusted using the Benjamini–Hochberg procedure. Standardized effect sizes (Cohen’s d) and 95% confidence intervals (CIs) of the differences are reported in the Results section to quantify the magnitude and uncertainty of the observed effects.

### 2.5. Architecture and Parameter Settings of the SE-EEG-Inception Model

The proposed SE-EEG-Inception model was implemented using TensorFlow 2/Keras and trained on a workstation equipped with an Intel CPU and an NVIDIA GPU. Network weights were initialized using the He normal method. To ensure full computational reproducibility, the complete architectural details—including tensor transformations, padding, stride, filter counts, activation functions, normalization layers, dropout rates, pooling dimensions, SE reduction ratio, and total parameter counts—are comprehensively detailed in Supplementary Table S1. The model was designed to integrate multi-domain EEG feature representation with multi-scale convolutional learning and channel-wise attention [18]. In this study, the input feature matrix was constructed from time-domain, frequency-domain, and spatial-domain EEG features extracted from selected sensorimotor-related channels. Compared with directly using raw EEG signals, this representation provides explicit neurophysiological information related to signal amplitude variation, Mu/Beta rhythm modulation, spectral complexity, and inter-channel spatial coupling.

**Figure 6:**
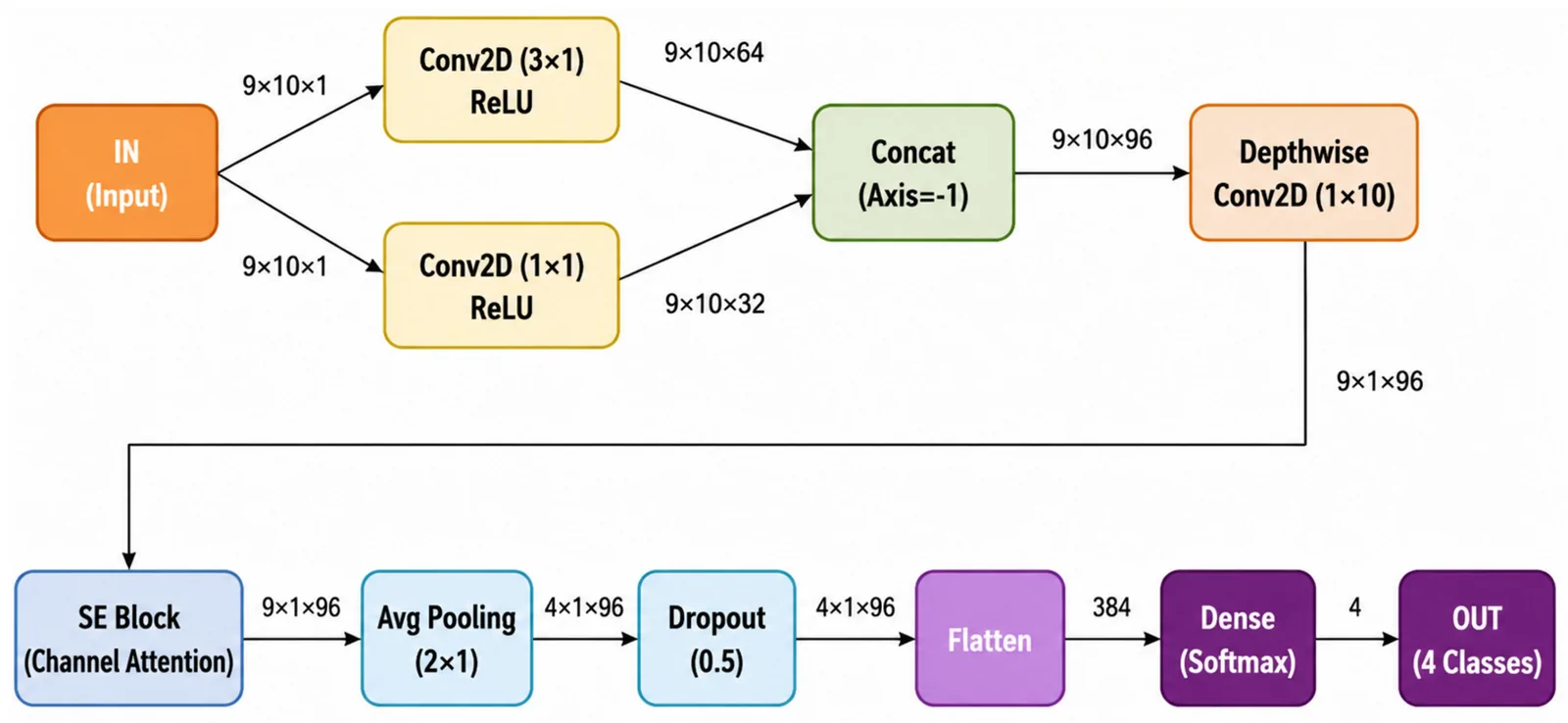
Architecture of the proposed SE-EEG-Inception model.

Based on this fused feature representation, the EEG-Inception backbone was used to extract complementary features at multiple scales. Specifically, parallel convolutional branches with different kernel sizes were adopted to act as localized feature combiners across the heterogeneous feature axis. The 3 × 1 convolution branch was used to learn cross-feature combinations along the heterogeneous feature axis, while the 1 × 1 convolution branch was used to refine intra-channel feature representations and reduce redundant information. In addition, a depthwise convolution branch was introduced to capture local synergistic activations among EEG channels. The outputs of different branches were then compressed by average pooling and concatenated to form a fused multi-scale representation, which was subsequently fed into the classification layers, as shown in Figure 6.

The feature extraction process of the (k)-th convolutional branch can be formulated as:

(17) $$Z_{k}=\phi \left(W_{k}*F+b_{k}\right),\;k=1,2,\ldots ,N$$

where ∗ denotes the convolution operation, $W_{k}$ is the convolution kernel of the k-th branch, N is the number of parallel convolution branches, and ϕ(·) is the nonlinear activation function.

To further enhance discriminative feature learning at the channel level, an SE module was embedded into the EEG-Inception architecture [19]. The SE module consists of three operations: squeeze, excitation, and feature recalibration. In the squeeze operation, global average pooling is applied to the input feature maps to obtain global statistical information for each channel:

(18) $$z_{c}=\frac{1}{H\times W}\sum_{i=1}^{H}\sum_{j=1}^{W}u_{c}\left(i,j\right)$$

where $u_{c}\left(i,j\right)$ represents the feature response of channel c at spatial position $\left(i,j\right)$, and $z_{c}$ is the global channel descriptor obtained by global average pooling.

In the excitation operation, a two-layer fully connected network is used to learn channel-wise attention weights:

(19) $$s=\sigma \left(W_{2}\delta \left(W_{1}z\right)\right)$$

where δ(∙) denotes the ReLU activation function, σ(∙) denotes the Sigmoid function, and s is the learned channel attention weight vector.

Finally, the learned attention weights are applied to the original feature maps through channel-wise multiplication:

(20) $${\tilde{u}}_{c}=s_{c}\cdot u_{c}$$

where $s_{c}$ denotes the attention weight of channel c, $u_{c}$ is the original feature map of channel c, and ${\tilde{u}}_{c}$ is the recalibrated channel feature.

**Figure 7:**
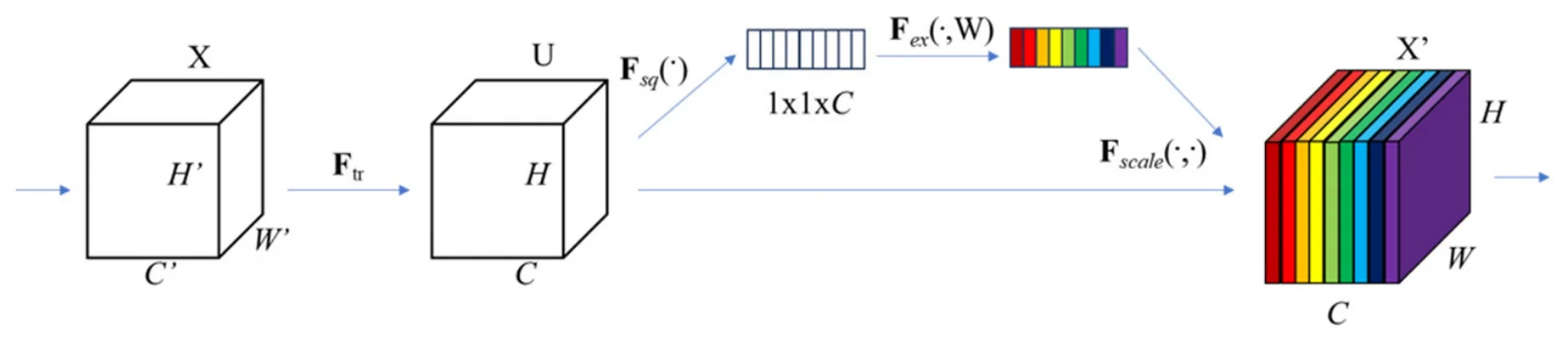
Structure of the Squeeze-and-Excitation (SE) module.

To allow the network to selectively emphasize informative feature maps, a Squeeze-and-Excitation (SE) block was incorporated. The specific internal operations of this channel-wise feature recalibration are depicted in Figure 7. The resulting channel-attention weights implement a data-driven feature re-weighting mechanism, scaling the feature responses based on their relative predictive utility for the classification task. This architectural design enables SE-EEG-Inception to capture discriminative patterns across different motor imagery categories.

To address inter-subject variability and feature redundancy in EEG signals, a targeted training and validation strategy was employed [20,21]. The main objective was to enable the proposed SE-EEG-Inception model to learn discriminative motor imagery representations while maintaining stable generalization performance.

Given the relatively small sample size inherent to EEG datasets, strict regularization strategies were embedded within the SE-EEG-Inception architecture to prevent overfitting and ensure model generalization. Rather than employing external data augmentation, the model relies on structural constraints. Specifically, Spatial Dropout layers with a rate of 0.3 were applied after pooling operations, and a standard Dropout rate of 0.35 was applied before the final classification layer. Additionally, Batch Normalization was utilized after every convolutional operation to stabilize the internal covariate shift. To further penalize overly complex weights, L2 weight decay (regularization factor = 4 × 10^−5^) was applied to all convolutional and dense layers. Finally, an early stopping mechanism (patience = 35 epochs) was utilized during training to halt the process when the validation loss ceased to improve, ensuring the optimal model weights were retained.

**Figure 8:**
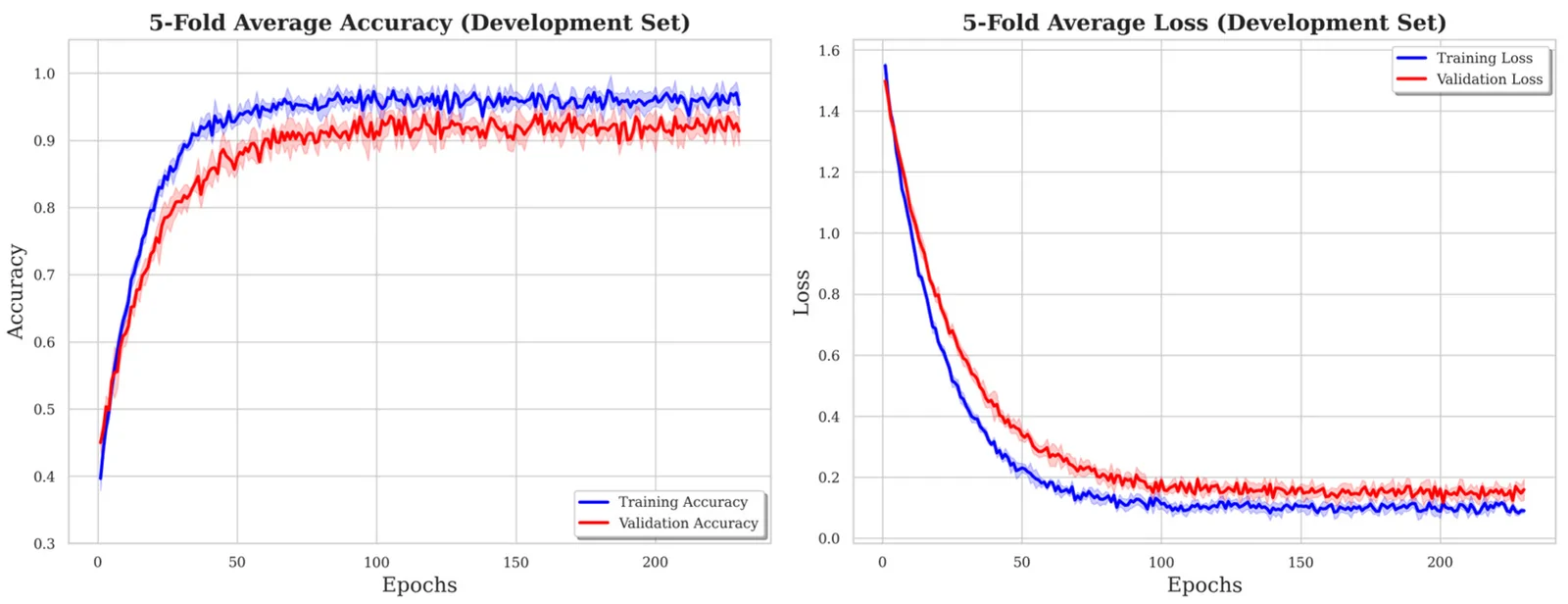
Training and validation accuracy/loss curves.

The model was optimized using the Adam optimizer with an initial learning rate of 0.00012. L2 regularization with a coefficient of (4 × 10^−5^) was applied to the convolutional and dense layers to reduce overfitting. The initial learning rate was determined through grid search to balance convergence speed and late-stage parameter refinement. The batch size was set to 32 to satisfy GPU memory constraints while maintaining stable gradient estimation. To alleviate potential bias caused by class imbalance, class weights were computed from the training labels and incorporated into the training process. An early stopping strategy was adopted to prevent overfitting: training was terminated when the validation loss did not improve for 35 consecutive epochs, and the best-performing weights were restored. In addition, when the validation loss plateaued for 12 consecutive epochs, the learning rate was multiplied by 0.6 to enable finer parameter adjustment. All hyperparameters and model performance were evaluated under a five-fold cross-validation framework to ensure robustness and reproducibility. As shown in Figure 8, both training and validation accuracy gradually increased while the corresponding losses decreased during optimization, indicating that the model achieved stable convergence without apparent overfitting. The validation performance remained consistent with the training process, demonstrating the effectiveness of the adopted optimization strategy.

The categorical cross-entropy loss function was used for model optimization:

(21) $$L_{CE}=-\frac{1}{N}\sum_{i=1}^{N}\sum_{k=1}^{K}y_{i,k}log\left({\hat{y}}_{i,k}\right)$$

where N is the number of samples, K is the number of classes, $y_{i,k}$ is the one-hot encoded ground-truth label, and ${\hat{y}}_{i,k}$ is the predicted probability for class k.

### 2.6. Data Partitioning and Validation Design

To evaluate the model’s personalized decoding capability, a subject-dependent (intra-subject) evaluation strategy was employed [22,23]. For each of the 292 participants, their EEG trials were individually partitioned. A standard five-fold cross-validation protocol was performed within each participant’s data. Specifically, 80% of the trials from a single subject were used for training and validation, and the remaining 20% were used for testing. The final performance metrics were calculated by averaging the test results across all 292 individually trained models, providing a strictly unbiased evaluation of the model’s personalized generalization capability. To ensure absolute methodological transparency, a comprehensive participant-flow and data-partitioning diagram is presented in Figure S1.

To account for the participant-level class imbalance introduced during the artifact rejection phase, Balanced Accuracy and Cohen’s Kappa were incorporated as primary evaluation metrics alongside Sensitivity, Specificity, Precision, and the macro-averaged F1-score. To provide a comprehensive diagnostic breakdown, these metrics were calculated on a per-class basis and aggregated across all participants from their respective intra-subject test sets. Furthermore, for the binary classification tasks (i.e., left- versus right-arm imagery, and left- versus right-leg imagery), separate binary models were not trained. Instead, to explicitly evaluate the model’s discriminative capability on symmetric limbs, these binary performance metrics were derived directly by restricting the cross-validated predictions of the primary subject-independent four-class model to the relevant specific task subsets. All performance metrics are reported with their respective 95% Confidence Intervals (CIs) to transparently quantify statistical uncertainty.

## 3. Results

### 3.1. Temporal Feature Extraction of Motor Imagery EEG

**Figure 9:**
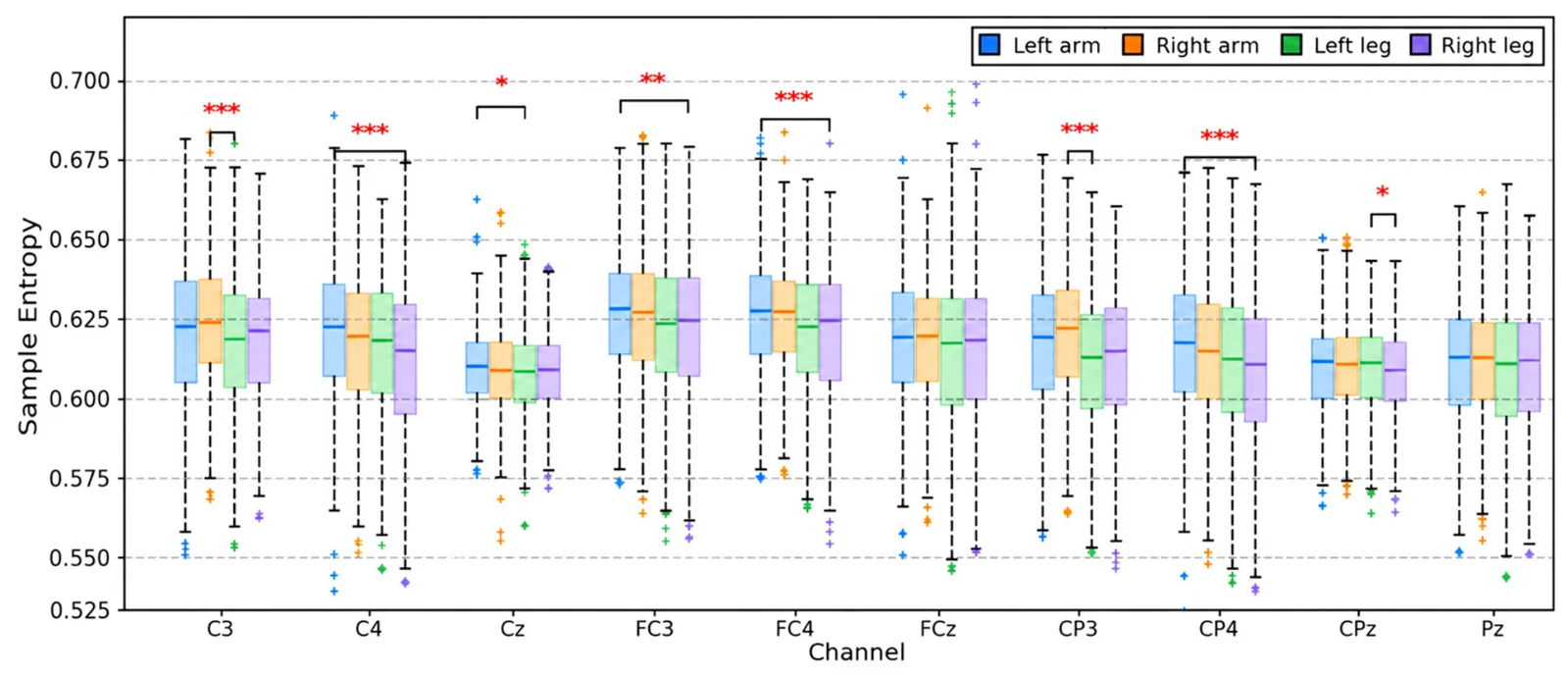
Distribution of sample entropy across selected EEG channels. Note: The plots illustrate the raw feature distributions fed into the classifier. To prevent pseudoreplication, the statistical significance markers (* $p_{FDR}$ < 0.05, ** $p_{FDR}$ < 0.01, *** $p_{FDR}$ < 0.001) represent FDR-corrected post hoc tests calculated strictly at the participant level (*n* = 292). The + symbol represents the mean value of the corresponding group.

Figure 9 shows the distribution of sample entropy across the ten selected motor-related EEG channels under the four MI tasks. Overall, the sample entropy values were mainly concentrated between 0.600 and 0.640, indicating that the temporal complexity of EEG signals during MI remained within a relatively stable range while still showing task-dependent differences. The left- and right-arm imagery tasks exhibited higher sample entropy values in sensorimotor-related channels such as C3, C4, FC3, and FC4, with median values mostly ranging from 0.623 to 0.629. In contrast, the left- and right-leg imagery tasks showed slightly lower values in these channels, with median values mainly distributed between 0.615 and 0.625, suggesting differences in EEG temporal complexity between arm and leg imagery.

At the channel level, FC3 and FC4 showed relatively high sample entropy values, with the main distributions of the four tasks ranging approximately from 0.606 to 0.640, indicating that fronto-central channels exhibited distinct task-dependent variance in temporal complexity. In CP3 and CP4, the median sample entropy values of arm imagery were approximately 0.615–0.622, whereas those of leg imagery were approximately 0.611–0.615, showing clearer task-dependent differences. By comparison, Cz, CPz, and Pz showed more concentrated distributions, with median values mostly around 0.609–0.613, indicating relatively weaker class separability in these midline channels. The significance analysis further showed clear inter-task differences in C3, C4, FC3, FC4, CP3, and CP4, suggesting that sample entropy can reflect task-dependent variations in EEG temporal complexity and has discriminative value for multi-class MI-EEG classification, particularly in central and centro-parietal channels.

### 3.2. Frequency-Domain Feature Extraction of Motor Imagery EEG

**Figure 10:**
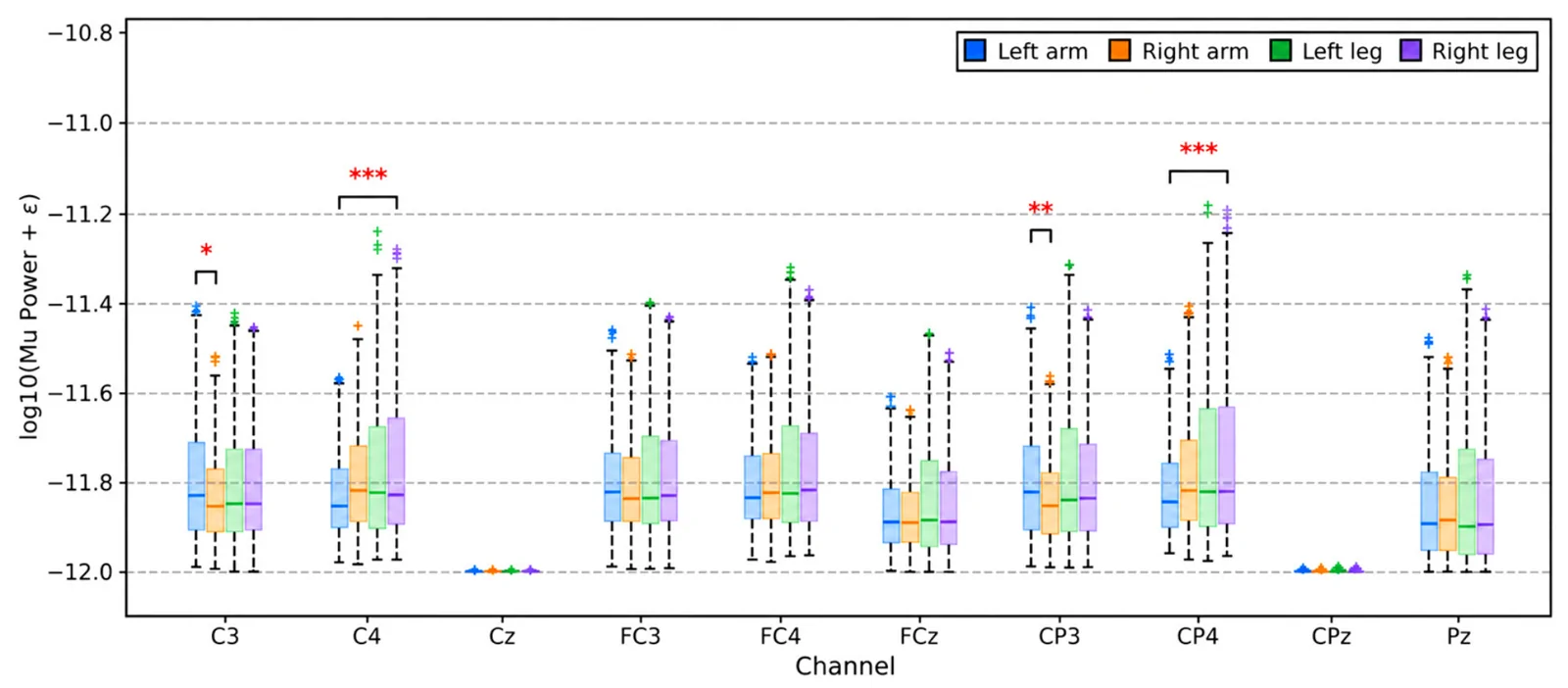
Mu power distributions across selected EEG channels. Note: The plots illustrate the raw feature distributions fed into the classifier. To prevent pseudoreplication, the statistical significance markers (* $p_{FDR}$ < 0.05, ** $p_{FDR}$ < 0.01, *** $p_{FDR}$ < 0.001) represent FDR-corrected post hoc tests calculated strictly at the participant level (*n* = 292). The + symbol represents the mean value of the corresponding group.

**Figure 11:**
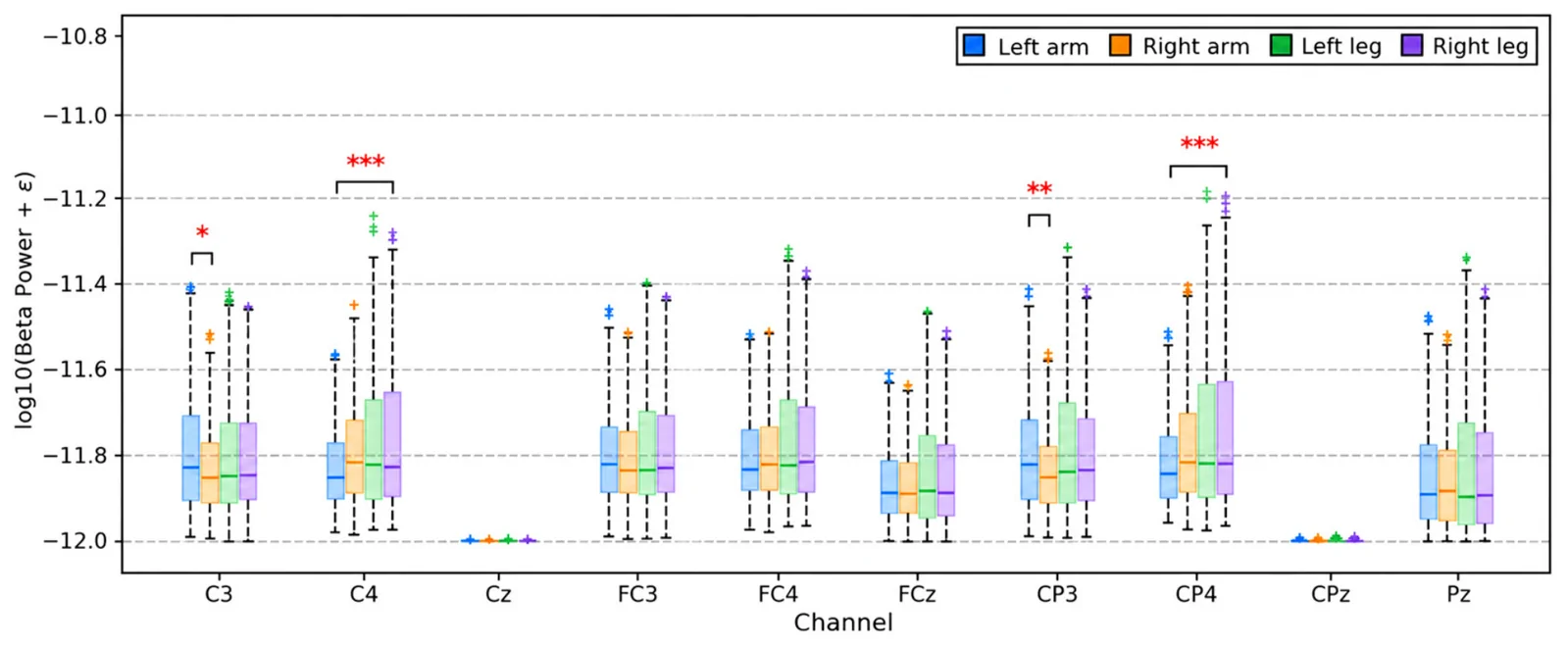
Beta-band power distributions across selected EEG channels. Note: The plots illustrate the raw feature distributions fed into the classifier. To prevent pseudoreplication, the statistical significance markers (* $p_{FDR}$ < 0.05, ** $p_{FDR}$ < 0.01, *** $p_{FDR}$ < 0.001) represent FDR-corrected post hoc tests calculated strictly at the participant level (*n* = 292). The + symbol represents the mean value of the corresponding group.

Figure 10 and Figure 11 show the distributions of Mu and Beta-band power across the ten selected EEG channels under the four MI tasks. In the Mu band, left-arm imagery exhibited higher power in C3, FC3, and CP3, with median values of approximately −12.32, −12.29, and −12.29, respectively. Right-arm imagery showed higher power in C4 and CP4, with median values of approximately −12.28 and −12.28, respectively. In comparison, the power values of left- and right-leg imagery in these channels were generally between those of the left- and right-arm tasks, although relatively high Mu power was also observed in FC4 and CP4. In particular, the median value of right-leg imagery in FC4 was approximately −12.28.

Task-dependent differences in Mu power were more evident in C3, C4, CP3, and CP4. In C3, left-arm imagery showed higher Mu power than right-arm imagery ($p_{FDR}$ < 0.001, Cohen’s d = 0.718, 95% CI: 0.251 to 0.346) and leg imagery ($p_{FDR}$ < 0.01, Cohen’s d = 0.195, 95% CI: 0.035 to 0.136), whereas in C4, right-arm imagery showed higher Mu power than left-arm imagery. In CP3, left-arm imagery showed higher power than right-arm imagery, while in CP4, right-arm and leg imagery exhibited higher power levels. These results indicate that different MI tasks induced distinct Mu rhythm modulation patterns across sensorimotor-related channels, with a particularly clear channel-dependent difference between left- and right-arm imagery.

In the Beta band, task-dependent differences were also mainly observed in C3, C4, FC3, FC4, CP3, and CP4. Left-arm imagery exhibited higher Beta power in C3, with a median value of approximately −12.71, whereas right-arm imagery showed higher Beta power in C4 and FC4, with median values of approximately −12.70 and −12.57, respectively. Left- and right-leg imagery showed relatively prominent Beta power in FC3, FC4, and CP4; for example, the median values of right-leg imagery in FC3 and CP4 were approximately −12.60 and −12.72, respectively. Compared with the Mu band, the differences between arm and leg imagery in the Beta band were more concentrated in fronto-central and centro-parietal channels, suggesting that Beta power provides complementary frequency-domain information for distinguishing different limb-related MI tasks.

### 3.3. Spatial Feature Extraction of Motor Imagery EEG

**Figure 12:**
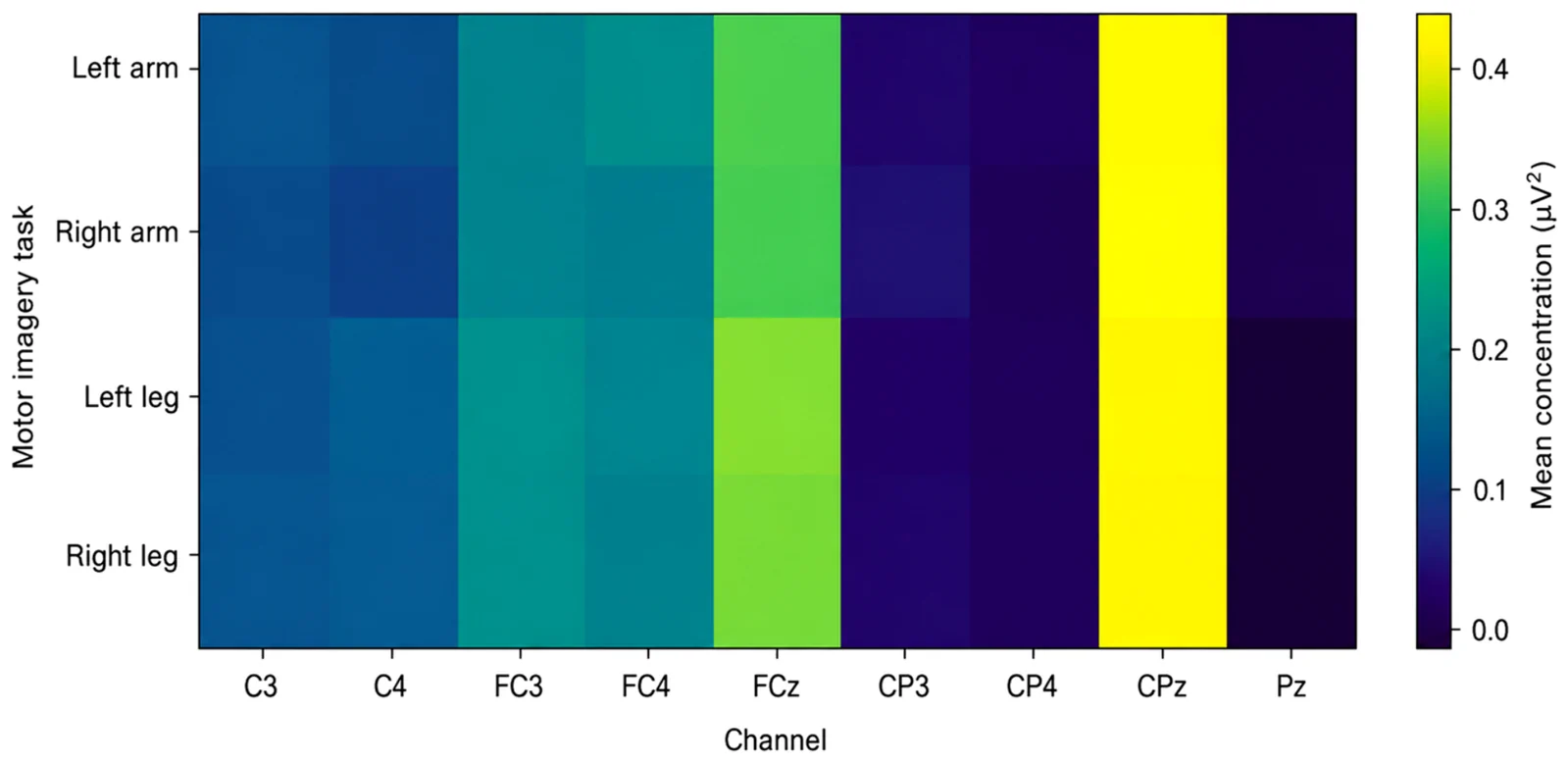
Spatial feature comparison across trials.

Figure 12 shows the distribution of the mean correlation between each selected channel and Cz under the four MI tasks. A higher correlation coefficient indicates stronger signal correlation between the corresponding channel and the central sensorimotor channel Cz. Overall, CPz showed the strongest correlation with Cz across all tasks, with mean correlation coefficients ranging from approximately 0.412 to 0.441. Specifically, the values for left-arm and right-arm imagery were approximately 0.441 and 0.439, respectively, whereas those for left-leg and right-leg imagery were approximately 0.412, indicating a strong synchronized variation between CPz and Cz during MI tasks.

Task-dependent differences were also observed across channels. Left and right-arm imagery showed higher correlations in CPz, whereas left and right-leg imagery exhibited relatively stronger correlations in FCz and FC3. Specifically, the mean correlations in FCz were approximately 0.353 and 0.345 for left-leg and right-leg imagery, respectively, which were higher than those for left-arm and right-arm imagery, approximately 0.307 and 0.306. In FC3, the correlations for left-leg and right-leg imagery were approximately 0.207 and 0.201, respectively, also higher than those for left-arm and right-arm imagery, approximately 0.178 and 0.179. These results suggest that leg imagery is associated with distinct signal correlation patterns in fronto-central regions.

In contrast, CP3, CP4, and Pz showed relatively low correlations with Cz. The mean correlation coefficients of CP3 and CP4 were mostly close to 0–0.03 across tasks, while Pz showed slightly negative correlations during left-leg and right-leg imagery, approximately −0.036 and −0.030, respectively. These findings indicate that different MI tasks not only differ in local rhythm modulation but also produce distinct inter-channel signal correlation patterns. Among the selected channels, CPz, FCz, and FC3 provided relatively strong spatial discriminative information for distinguishing limb-related MI tasks.

### 3.4. Performance Evaluation of the SE-EEG-Inception-Based Motor Imagery Task Classification Model

All compared baseline models were evaluated using the identical data partitioning strategy, internal five-fold cross-validation protocol, and evaluation metrics as the proposed SE-EEG-Inception model (as explicitly detailed in Section 2.6). The primary evaluation focused on the intra-subject setting, where data were strictly partitioned within each participant to train personalized models. Data augmentation was applied only to the training set after data partitioning to avoid information leakage. Classification accuracy and macro-averaged F1-score were used as the main evaluation metrics, and the results are reported as mean ± standard deviation over five folds [24].

To evaluate the performance of the proposed SE-EEG-Inception model, several representative baseline models were selected for comparison, encompassing strong classical BCI pipelines (e.g., Filter-Bank Common Spatial Pattern (FBCSP) [25,26], Riemannian Geometry Tangent Space Classifier (TSC) [27], and SVM) and contemporary deep learning architectures (e.g., Transformer, EEGNet, CNN, ACNet, MSTGCN). To ensure fair comparisons and avoid disadvantaging models designed for raw signals, contemporary raw-signal networks (such as EEGNet and temporal CNN) were trained directly on raw filtered EEG time-series [28], whereas feature-based models utilized our engineered multi-domain matrix. All baselines were evaluated using identical data partitions and equivalent computational tuning budgets. Specifically, classical models were optimized via grid search (e.g., SVM regularization C in [0.1, 100]), while deep learning baselines were tuned for network capacities and dropout rates (0.25–0.5) under identical early-stopping constraints. ACNet provides a typical reference for channel-attention mechanisms, and MSTGCN represents graph-convolution-driven spatial modeling, helping to complete our comparison across diverse technical paradigms.

As shown in Table 2, SE-EEG-Inception achieved higher classification accuracy and macro-F1 scores than all baseline models under both sample-level and subject-level classification settings. These results suggest that integrating multi-scale convolution, channel-wise attention, and multi-domain EEG feature representation can improve the discriminative capacity and generalization performance of MI-EEG decoding.

**Figure 13:**
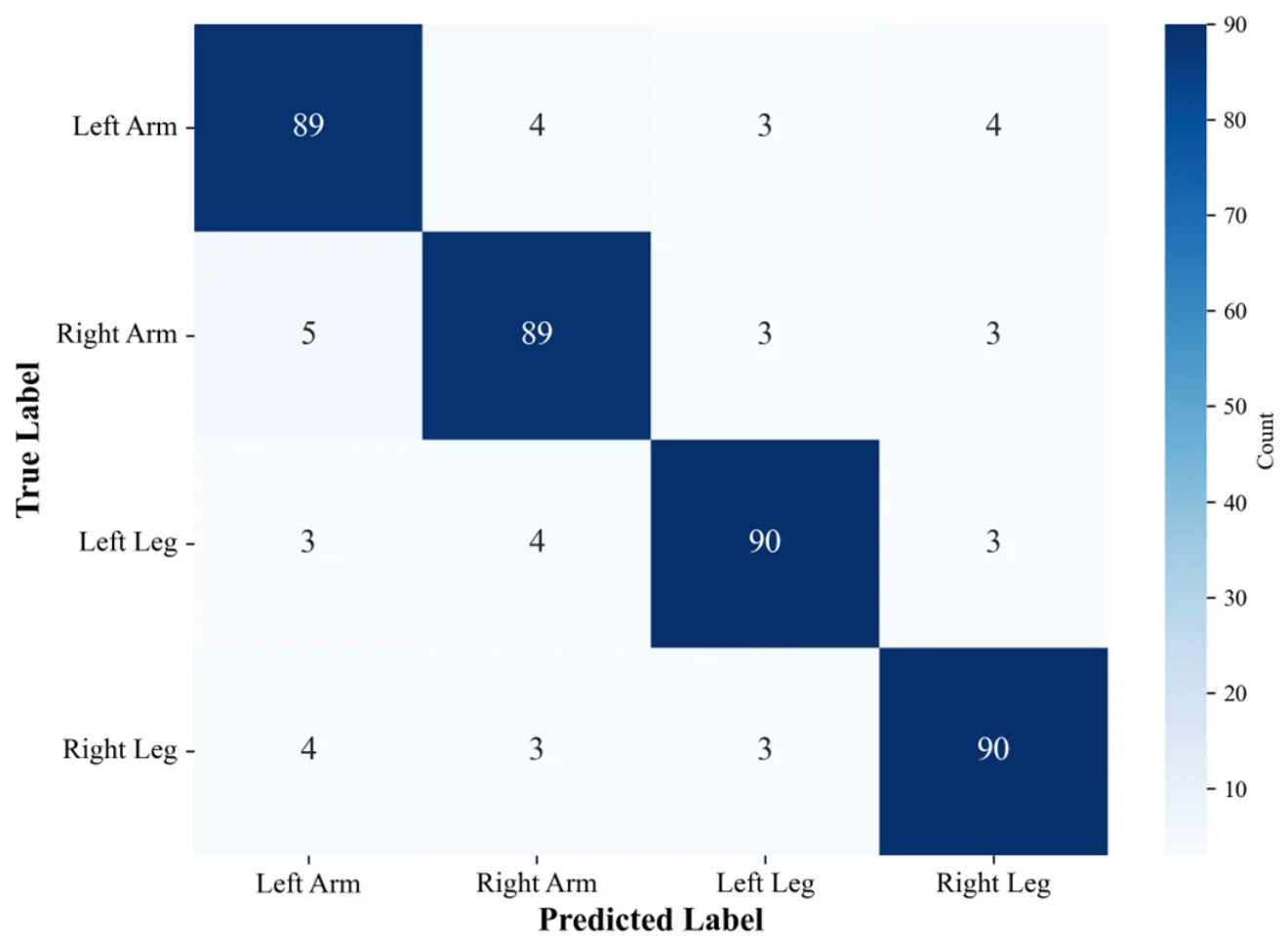
Aggregated four-class confusion matrix.

To provide a detailed diagnostic analysis of the SE-EEG-Inception model, a four-class confusion matrix aggregated across all cross-validation folds is presented in Figure 13. To account for slight class imbalances post-artifact rejection, both Balanced Accuracy and Cohen’s Kappa were evaluated and found to align closely with the high overall intra-subject classification accuracy, demonstrating strong discriminative performance across all classes. Comprehensive class-specific diagnostic metrics (including Sensitivity, Specificity, Precision, and F1-scores with their respective 95% CIs) are detailed in Supplementary Table S2 for brevity. Additionally, by restricting our four-class predictions to the binary subsets, the model demonstrated an 88.3% accuracy for the upper-limb binary classification (Left vs. Right Arm) and a 90.0% accuracy for the lower-limb binary classification (Left vs. Right Leg).

Crucially, an analysis of the performance distribution demonstrates robust personalized decoding across the entire user base, with the model effectively adapting to individual EEG characteristics to achieve a consistent mean intra-subject accuracy of 89.4%. The evaluation confirms that the proposed SE-EEG-Inception architecture reliably captures subject-specific motor imagery patterns, demonstrating stable performance and strong individual adaptability across the 292 participants.

To evaluate the interpretability of the SE-EEG-Inception model without overstating mechanistic inferences, we conducted a post hoc analysis of the spatial kernel weights extracted directly from the DepthwiseConv2D layer. Unlike feature-map attention (e.g., SE blocks) which operates on abstract latent channels, the spatial depthwise convolution directly aggregates signals across the distinct EEG electrodes, making its learned weights physically meaningful. The distribution of the mean absolute kernel weights across the 10 EEG electrodes (Figure S2) reveals that the model effectively prioritized physiologically relevant regions. Specifically, higher predictive weights were localized to the sensorimotor channels (e.g., C3, C4, CP3, CP4), which correspond to the primary motor cortex responsible for upper and lower limb imagery. While this confirms that the network utilizes expected contralateral and overlapping spatial patterns for classification, these weights represent data-driven predictive importance and should not be interpreted as direct source-level neurophysiological mapping.

Furthermore, to explicitly quantify the alleviation of inter-subject variability, the dispersion of participant-level accuracies was evaluated. The proposed SE-EEG-Inception model exhibited a remarkably narrow Interquartile Range (IQR = 3.75%) and Standard Deviation (SD = 4.13%) across the tested subjects, compared to the standard temporal CNN baseline (IQR = 8.59%, SD = 5.37%). This distinct quantitative reduction in performance dispersion confirms that the proposed architecture, coupled with domain adaptation, effectively mitigates cross-subject variance.

### 3.5. Ablation Study

A comprehensive ablation experiment was conducted to quantitatively evaluate the independent contribution of the core components in the proposed framework. The evaluated variants included the removal of: (1) the SE attention module, (2) the multi-scale architecture, (3) time-domain features, (4) frequency-domain features, and (5) spatial correlation features. Performance was measured via participant-level mean intra-subject accuracy.

**Figure 14:**
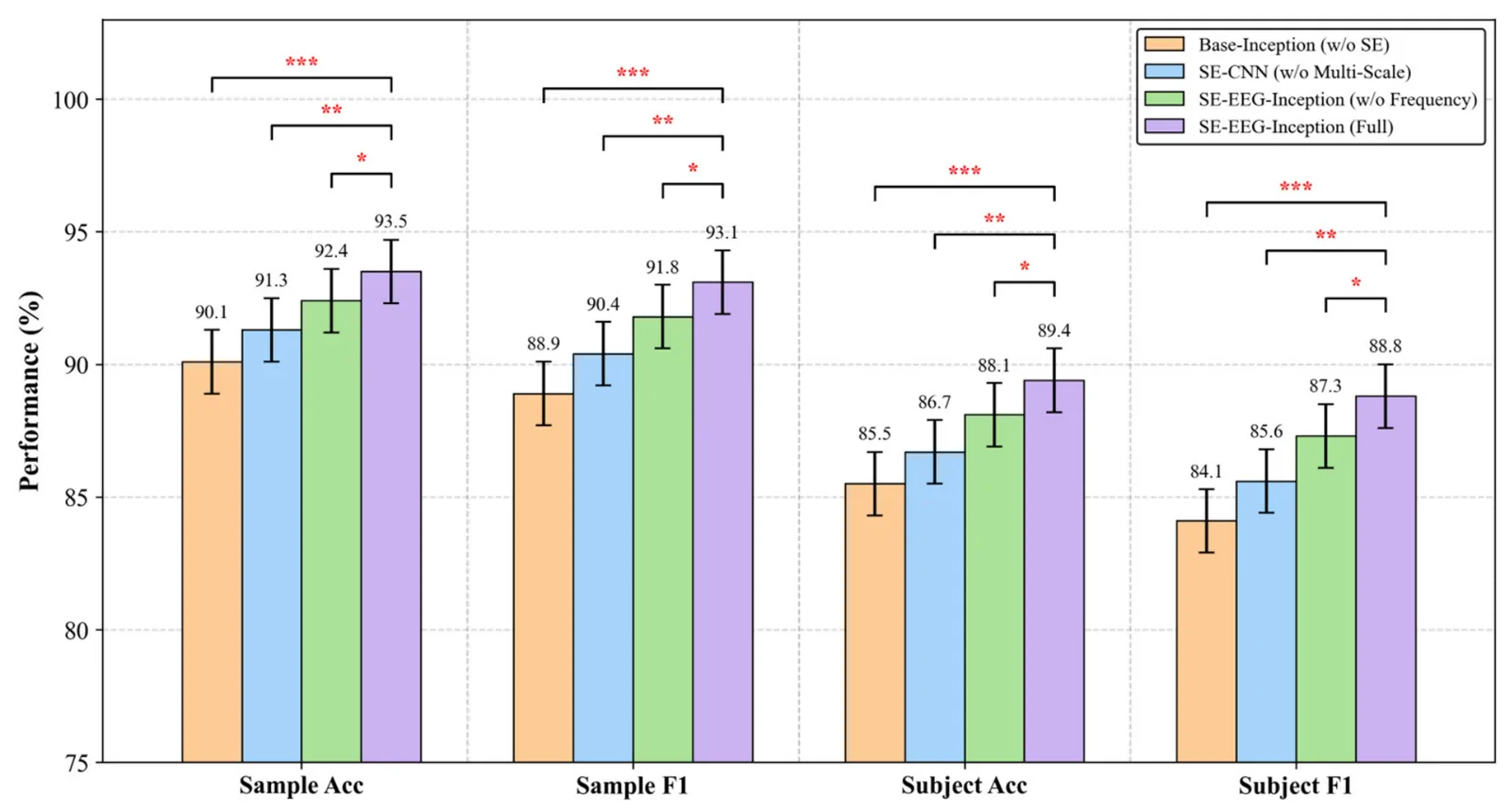
Performance comparison of ablation study. Note: We have added explanations for *, ** and *** in the figure caption. The markers represent statistical significance levels: * for (*p* < 0.05), ** for (*p* < 0.01), and *** for (*p* < 0.001). All changes are tracked.

As illustrated in Figure 14, the empirical results reveal nuanced module contributions. The removal of the SE attention module caused the most severe performance degradation (mean intra-subject accuracy dropping from 89.4% to 85.5%). Similarly, removing the multi-scale architecture reduced the mean accuracy to 86.7%, and ablating the frequency-domain features reduced it to 88.1%. These findings confirm their critical roles in robust personalized decoding. These findings suggest that while spatial-spectral representations coupled with channel attention are essential, the temporal features and multiscale convolutional branches may offer redundant information in this specific paradigm. For future cross-subject BCI deployments, a streamlined, single-scale architecture may prevent over-parameterization and further enhance generalization.

Furthermore, to determine whether the local multiscale convolutional structure captures substantive meaning across the heterogeneous feature axis, a sensitivity analysis was conducted by randomly permuting the order of the 9 feature rows during the test phase. Across five random permutations, the model’s blind-test accuracy exhibited a significant degradation, dropping significantly from the optimal baseline. This substantial performance drop empirically validates that the 3 × 1 local convolutions do not merely act as global feature mixers, but actively capture and rely upon complex, substantive non-linear relationships (covariances) among adjacent features in the predefined matrix. This confirms that the claimed local structure has substantive predictive meaning for the decoding task.

## 4. Discussion

It should be explicitly acknowledged that this study is fundamentally an offline proof-of-concept conducted using laboratory-grade EEG data from healthy, seated participants. Consequently, the present findings do not provide direct technical support for real-world intelligent prostheses or neurorehabilitation applications.

Evaluated under a rigorous intra-subject cross-validation protocol, the proposed model achieved a mean participant-level 4-class accuracy of 89.4%, with binary classification accuracies of 88.3% for left- versus right-arm imagery and 90.0% for left- versus right-leg imagery across the four motor imagery (MI) tasks. These quantitative results demonstrate that the proposed method achieves high-accuracy classification using non-invasive EEG signals. These results are meaningful not only in terms of classification performance but also in demonstrating the feasibility of non-invasive EEG for relatively fine-grained classification of signals recorded during instructed motor imagery. Compared with more subtle movements such as finger or facial imagery, large-limb movements have more distinguishable somatotopic representations in the sensorimotor cortex, which provides a suitable basis for evaluating model generalization in multi-class and multi-limb MI scenarios. More importantly, symmetric limbs, such as the left and right arms or the left and right legs, perform the same type of imagined movement and may therefore evoke highly similar neural activation patterns. The present results suggest that, through the coordinated use of multi-scale feature extraction and channel-wise attention, the model can capture subtle but stable neural differences between symmetric limb movements. This finding supports the development of multi-degree-of-freedom MI-BCI systems, particularly for applications such as intelligent prosthetic control, exoskeleton assistance, and neurorehabilitation training, where accurate classification of EEG signals from multiple limb-related motor intentions is required.

Furthermore, our multi-domain feature analysis revealed distinct spectral and spatial patterns between ipsilateral upper- and lower-limb conditions (e.g., left-arm versus left-leg imagery). Because the primary objective of this study was to evaluate holistic multi-limb MI decoding performance rather than to conduct a definitive neurophysiological mapping among different effectors, these ipsilateral variations are strictly presented as exploratory observations. Future studies incorporating separate, dedicated baseline-normalized comparisons are required to fully elucidate these specific inter-limb neurophysiological differences.

This study included participants from two distinct recruitment sites, yielding a combined dataset of 242 college students and 50 community-dwelling older adults. We explicitly note that the primary objective of pooling these datasets was not to investigate age-related neurophysiological differences or to formally establish a cross-age generalization framework. Rather, the combination of these cohorts was intended purely to introduce real-world data heterogeneity—encompassing natural variations in environmental settings, baseline impedance, and trial counts (58 versus 82 trials per task). Evaluating the SE-EEG-Inception model on this pooled, heterogeneous dataset effectively demonstrates its robust capability to extract personalized discriminative multi-domain features from signals recorded during instructed motor imagery. Although spectral variations were observed across different demographic cohorts and task conditions, these patterns are interpreted strictly as discriminative spectral signatures utilized by the machine learning model. In the absence of a pre-task baseline, mechanistic inferences regarding neural synchronization or desynchronization dynamics are avoided.

Formally disentangling the confounding effects of recruitment site, acquisition structure, and age cohort falls outside the primary algorithmic scope of this current study [29]. Consequently, rigorous leave-cohort-out validation and independent statistical testing of performance differences between these specific demographic groups remain a crucial avenue for our subsequent research.

Several limitations should be acknowledged, with a primary focus on threats to internal validity. First, regarding the experimental paradigm, the continuous presentation of visual cues during the imagery period introduces a potential confounding risk of visually evoked activity. Although visual stimuli were strictly matched for physical attributes and the analysis was constrained to the 8–30 Hz sensorimotor band, we did not employ a dedicated cue-only control condition or restrict decoding strictly to a post-cue temporal window. Second, methodological constraints exist within the evaluation pipeline: hyperparameter tuning was conducted without a strictly nested cross-validation framework, which may introduce optimistic bias, and the single-session design involves inherent intra-subject trial dependence, leaving the model’s cross-session reliability unevaluated. Third, the current validation is strictly offline; real-time operational performance, user adaptation dynamics, and closed-loop BCI integration remain to be evaluated. Furthermore, the mechanistic interpretations of the SE attention weights reflect data-driven feature calibration rather than direct physiological suppression of noise or redundancy.

As secondary constraints regarding external generalizability, all EEG data were collected under controlled laboratory conditions, whereas real-world applications involve environmental noise, device movement, unstable electrode contact, and fluctuations in user attention [30,31]. Additionally, participants were healthy community-dwelling adults with limited geographic and demographic diversity; clinical populations with neurological disorders (e.g., stroke, Parkinson’s disease, Alzheimer’s disease) were not included. Exhaustive clinical covariates—such as handedness, detailed medication exposure, sleep status, and formalized cognitive screening—were not systematically recorded. Additionally, subjective task-related factors—such as self-reported imagery vividness, ease of execution, and preferred imagery modalities—were not collected. Future studies should incorporate structured interviews or validated questionnaires to quantify these individual cognitive characteristics and include them as covariates to better account for inter-individual and inter-group variability. Therefore, the present findings should not be directly generalized to clinical patient populations before future studies comprehensively document these clinical variables and conduct patient-specific validations.

Several critical online BCI performance metrics remain unevaluated in this offline framework, including information-transfer rate (ITR), end-to-end latency, calibration burden, computational complexity, online adaptation capabilities, session-to-session reliability, and asynchronous false-activation rates. The absence of a real-time control task further limits immediate operational generalization.

Furthermore, a central limitation of this study is the absence of continuous objective verification (e.g., surface EMG, accelerometry, or video monitoring) to ensure participants remained entirely motionless, despite the experiments being supervised by professional operators who immediately halted and restarted sessions upon observing overt movements. Although ICA was applied for artifact removal, the potential contribution of covert Muscle activation, minor postural adjustments, or subtle lateralized electromyographic contamination to the discriminative features cannot be definitively excluded. Therefore, the present results must be interpreted strictly as the classification of EEG signals recorded during instructed motor imagery, rather than the decoding of pure motor intentions independent of peripheral physiological correlates.

To address these limitations, future work will focus on three directions: lightweight and real-time model deployment using knowledge distillation, network pruning, and quantization; cross-dataset transfer learning to evaluate generalization across different acquisition devices, experimental paradigms, and electrode layouts; and clinical validation in patients with post-stroke motor dysfunction, spinal cord injury, or other motor impairments, thereby promoting the translation of MI-BCI technology from laboratory research to practical clinical applications.

It is important to acknowledge that the high classification accuracy and the extracted spatial convolution weights demonstrate the predictive utility of the model, but they do not constitute mechanistic neurophysiological explanations. In the absence of concurrent EMG monitoring to rule out micro-movements, and without high-density source localization, the interpretations of spatial coupling and electrode importance must remain strictly at the level of predictive feature mapping rather than causal biological inference.

## 5. Conclusions

In this study, the SE-EEG-Inception model achieved an average four-class motor imagery classification accuracy of 89.4% on an EEG dataset comprising 292 participants, with binary classification accuracies of 88.3% for left- versus right-arm imagery and 90.0% for left- versus right-leg imagery. The results indicate that non-invasive EEG can distinguish motor imagery patterns associated with different limbs and can also classify distinct predictive feature patterns between symmetric limbs, such as the left and right arms, when performing the same type of imagined movement. This capability is mainly supported by the coordinated use of multi-scale convolution and channel-wise attention: the former captures complementary multi-domain feature-channel representations, whereas the latter adaptively recalibrates channel weights based on predictive utility, thereby helping to alleviate feature redundancy and unequal channel contributions to achieve high personalized decoding accuracy. In addition, the fusion of time-domain, frequency-domain, and spatial-domain features further improves the separability of ambiguous categories. These findings suggest that MI-EEG decoding in highly heterogeneous populations is feasible and provide technical support for multi-limb BCI systems, particularly in applications such as intelligent prostheses and neurorehabilitation. Future work will focus on lightweight model design and real-time deployment, as well as further validation in clinical patient populations. Therefore, prospective online, cross-session, cross-device, and patient-specific clinical validations are absolute prerequisites before any practical translation or clinical deployment can be considered [32].

## Figures and Tables

**Table 1:** Processed dataset statistics.

Dataset	Young-Adult Cohort	Older-Adult Cohort
Confidentiality	Private	Private
Type (subtype)	MI	MI
#-Subjects	242	50
# Trials per task per subject	58	82
#-Sampling Rate	1000 Hz	1000 Hz
#-Channels	64	64
#-Duration	5 s	5 s
#-Trials	56,144	16,400
Tasks	Four-class MI	Four-class MI

**Notes:** The symbol # denotes “number of”.

**Table 2:** Performance comparison of baseline models and SE-EEG-Inception.

Model	Sample Acc (%)	Sample F1 (%)	Sample Recall (%)	Subject Acc (%)	Subject F1 (%)	Subject Recall (%)
FBCSP	73.5 ± 2.1	72.1 ± 2.2	71.8 ± 2.4	69.8 ± 2.5	68.2 ± 2.6	67.5 ± 2.4
Riemannian TSC	77.2 ± 1.8	75.8 ± 1.9	75.5 ± 2.0	73.1 ± 2.2	71.6 ± 2.3	70.4 ± 2.1
SVM	78.4 ± 1.5	77.2 ± 1.6	76.9 ± 1.7	74.5 ± 2.1	72.8 ± 2.2	71.8 ± 2.3
EEGNet	85.2 ± 1.4	83.8 ± 1.5	83.5 ± 1.5	80.6 ± 1.8	78.9 ± 1.9	78.2 ± 1.8
CNN	87.8 ± 1.2	86.5 ± 1.3	86.2 ± 1.4	82.9 ± 1.6	81.4 ± 1.7	80.9 ± 1.6
Transformer	89.5 ± 1.1	88.2 ± 1.2	88.0 ± 1.2	85.2 ± 1.5	84.1 ± 1.6	83.6 ± 1.5
ACNet	90.6 ± 0.9	89.4 ± 1.0	89.1 ± 1.0	86.4 ± 1.4	85.3 ± 1.5	84.8 ± 1.4
MSTGCN	91.5 ± 0.8	90.6 ± 0.9	90.3 ± 0.9	87.2 ± 1.2	86.5 ± 1.3	85.9 ± 1.3
**SE-EEG-Inception**	92.9 ± 0.6	92.5 ± 0.7	92.2 ± 0.7	89.4 ± 0.9	87.8 ± 1.0	87.4 ± 1.1

## Data Availability

The data presented in this study are not publicly available due to privacy protection and ethical restrictions related to human EEG recordings. The processed data, experimental materials, complete analysis code, fixed participant-level data splits, random seeds, anonymized feature matrices, and pretrained model weights may be made available from the corresponding author upon reasonable request and with permission from the relevant institution.

## Supplementary Materials

The following supporting information can be downloaded at: https://www.mdpi.com/article/10.3390/brainsci16090910/s1, Figure S1: Overview of data partitioning and workflow; Figure S2: Spatial electrode importance; Table S1: Complete architectural details and hyperparameters of the SE-EEG-Inception model; Table S2: Class-specific diagnostic performance metrics with 95% CIs.
