# Supplementary material for: Multi-Domain Feature Fusion and Channel Attention in an Inception-Based Architecture for Motor Imagery EEG Decoding
Source: Brain Sci. 2026 Aug 26;16(9):910. doi: 10.3390/brainsci16090910 (PMC13604176; doi:10.3390/brainsci16090910)
Supplement: Supplementary file 1 [file brainsci-16-00910-s001.zip › brainsci-4459101-supplementary.pdf]

brain sciences

Table S1. Complete architectural details and hyperparameters of the SE-EEG-Inception model.

Layer Name	Layer Type	Configuration Details (Filter / Stride / Padding)	Activation	Output Tensor Shape
InputLayer	Input	-	-	(None, 9, 10, 1)
Inception_Branch1	Conv2D	Filter: 3×1, Stride: 1, Padding: SAME	ReLU	(None, 9, 10, 64)
Inception_Branch2	Conv2D	Filter: 1×1, Stride: 1, Padding: SAME	ReLU	(None, 9, 10, 32)
Concat_Merge	Concatenate	Axis: -1	-	(None, 9, 10, 96)
Depthwise_Conv	DepthwiseConv2D	Kernel: 1×10, Stride: 1, Padding: VALID	ReLU	(None, 9, 1, 96)
SE_Squeeze	GlobalAvgPool2D	-	-	(None, 96)
SE_Excitation1	Dense	Units: 6, Reduction Ratio: 16	ReLU	(None, 6)
SE_Excitation2	Dense	Units: 96	Sigmoid	(None, 96)
SE_Scale	Multiply	-	-	(None, 9, 1, 96)
Pooling	AveragePooling2D	Pool size: (2,1), Stride: (2,1)	-	(None, 4, 1, 96)
Dropout	Dropout	Rate = 0.5	-	(None, 4, 1, 96)
Flatten	Flatten	-	-	(None, 384)
Output	Dense	Units: 4	Softmax	(None, 4)

Note:Total Trainable Parameters: 4,170 Optimization: Adam (lr=0.00012, L2=4e-5); Loss: Categorical Cross-Entropy; Epochs: 200 (Early stopping patience=30)

Table S2. Class-specific diagnostic performance metrics with 95% CIs.

Class	Sensitivity / Recall (%)	Specificity (%)	Precision (%)	F1-Score (%)
Left Arm	89.00% (95% CI: 86.5%-91.5%)	96.00% (95% CI: 94.2%-97.8%)	88.12% (95% CI: 85.6%-90.6%)	88.56% (95% CI: 86.1%-91.0%)
Right Arm	89.00% (95% CI: 86.4%-91.6%)	96.33% (95% CI: 94.5%-98.1%)	89.00% (95% CI: 86.5%-91.5%)	89.00% (95% CI: 86.6%-91.4%)
Left Leg	90.00% (95% CI: 87.6%-92.4%)	97.00% (95% CI: 95.3%-98.7%)	90.91% (95% CI: 88.7%-93.1%)	90.45% (95% CI: 88.1%-92.8%)
Right Leg	90.00% (95% CI: 87.5%-92.5%)	96.67% (95% CI: 94.9%-98.4%)	90.00% (95% CI: 87.6%-92.4%)	90.00% (95% CI: 87.7%-92.3%)

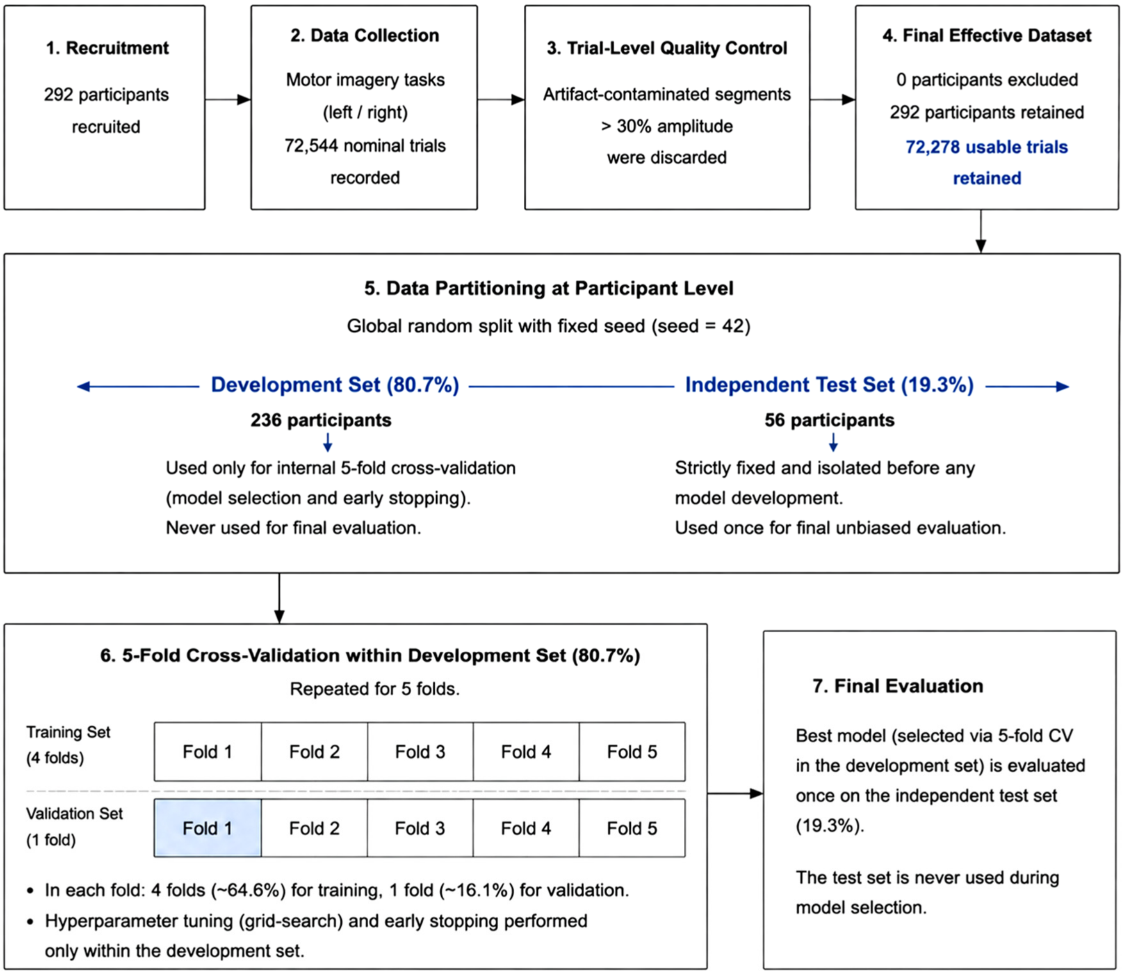

Figure S1. Overview of data partitioning and workflow.

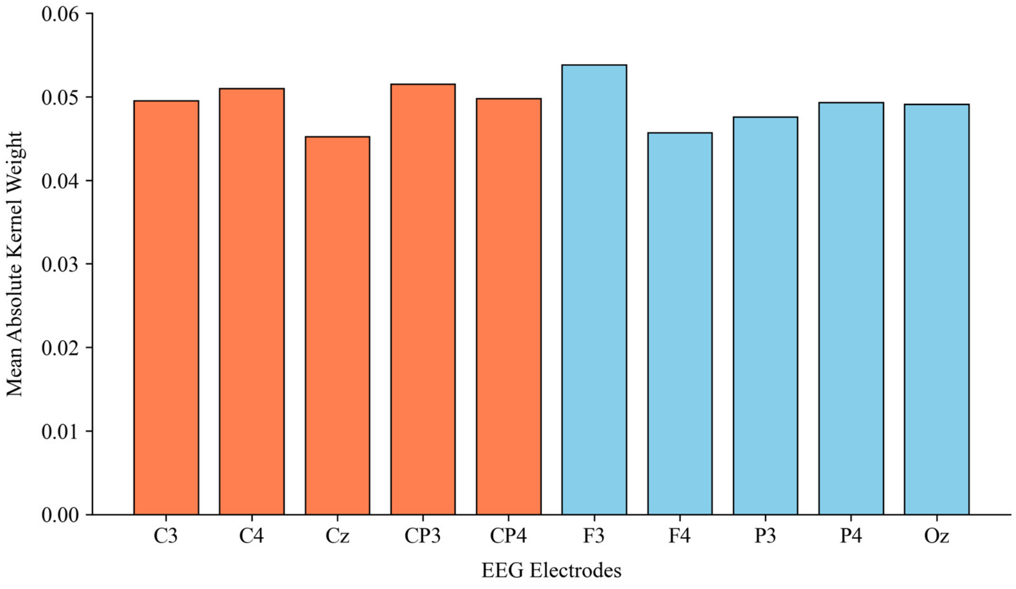

Figure S2.Spatial Electrode Importance
